# What are effective strategies for the implementation of care bundles on ICUs: a systematic review

**DOI:** 10.1186/s13012-015-0306-1

**Published:** 2015-08-15

**Authors:** Marjon J. Borgert, Astrid Goossens, Dave A. Dongelmans

**Affiliations:** Department of Intensive Care Medicine, Academic Medical Center, University of Amsterdam, PO Box 22660, 1100 DD Amsterdam, The Netherlands; Department of Quality Assurance and Process Innovation, Academic Medical Center, University of Amsterdam, PO Box 22660, 1100 DD Amsterdam, The Netherlands

## Abstract

**Background:**

Care bundles have proven to be effective in improving clinical outcomes. It is not known which strategies are the most effective to implement care bundles. A systematic review was conducted to determine the strategies used to implement care bundles in adult intensive care units and to assess the effects of these strategies when implementing bundles.

**Methods:**

The databases MEDLINE/PubMed, Ovid/Embase, CINAHL and CENTRAL were searched for eligible studies until January 31, 2015. Studies with (non)randomised designs on central line, ventilator or sepsis bundles were included if implementation strategies and bundle compliance were reported. Methodological quality was assessed by using the Downs and Black checklist. Data extraction and quality assessments were independently performed by two reviewers.

**Results:**

In total, 1533 records were screened and 47 studies were finally included. In 49 %, pre/post designs were used, 38 % prospective cohorts, and the remaining studies used retrospective designs (6 %), interrupted time series (4 %) and longitudinal designs (2 %). The methodological quality was classified as ‘fair’ in 77 %, and the remaining as ‘good’ (13 %) and ‘poor’ (11 %). The most frequently used strategies were education (86 %), reminders (71 %) and audit and feedback (63 %). Our results show that compliance is influenced by multiple factors, i.e. types and numbers of elements varied and different compliance measurements were reported. Furthermore, compliance was calculated within different time frames. Also, detailed information about compliance, such as numerators and denominators, was not reported. Therefore, recalculation of consistent monthly compliance levels was not possible.

**Conclusions:**

The three most frequently used strategies were education, reminders and audit and feedback. We conclude that the heterogeneity among the included studies was high due to the variety in study designs, number and types of elements and types of compliance measurements. Due to the heterogeneity of the data and the poor quality of the studies, conclusions about which strategy results in the highest levels of bundle compliance could not be determined. We strongly recommend that studies in quality improvement should be reported in a formalised way in order to be able to compare research findings. It is imperative that authors follow the standards for quality improvement reporting excellence (SQUIRE) guidelines whenever they report quality improvement studies.

**Electronic supplementary material:**

The online version of this article (doi:10.1186/s13012-015-0306-1) contains supplementary material, which is available to authorized users.

## Introduction

Because of the ageing population, the number of patients with chronic illnesses and comorbidities increases [1]. More complex medical care is needed for these patients when admitted to hospitals [1] of which the critically ill are admitted to the intensive care units (ICUs). To provide comprehensive care according to the best available evidence and to decrease the variation in daily care, clinical guidelines and protocols are developed [2]. Despite the efforts made in implementation, the adherence to guidelines and protocols is often poor [3], which negatively influences the quality of care [3, 4].

In order to encourage the adherence to clinical guidelines and to improve care processes, the Institute for Healthcare Improvement (IHI) has developed the concept of ‘care bundles’ [4–6]. Initially, care bundles were introduced to reorganise the structure and organisation of care processes within the ICU departments. For example, the central line bundle was developed to reduce bloodstream infections [5, 7]. Care bundles are designed around specific elements of patient care and consist of three to five key interventions, the so called elements [4]. These elements are either evidence based or are already generally accepted in ICUs and in national guidelines. The strength of a care bundle is that all elements must be performed in every eligible patient, unless medically contraindicated, using the all-or-none (AON) approach [4–6, 8].

The bundled approach has already proven to be effective in improving clinical outcomes [7, 9, 10]. In accordance with the model of Donabedian, high levels of bundle compliance should be achieved to improve clinical outcomes [11]. For instance, Resar et al. have shown that ICUs with the highest levels of bundle compliance had the highest rate of infection reduction [12]. Pronovost et al. demonstrated that the implementation of the central line bundle resulted in a large reduction in infection rates (up to 66 %) during the study period of 18 months [9]. Positive results can be obtained when improving the reliability of care processes to ensure patients receive all evidence-based interventions needed. This also includes the improvement of the organisational culture, i.e. the context in which care is delivered [13]. The IHI recommends achieving more than 95 % reliability [4]. Care bundles formed part of multiple patient safety initiatives in hospitals and ICUs worldwide and are nowadays widely accepted on ICUs.

Various strategies were described in the literature to encourage the implementation of care bundles on ICUs [14, 15]. Single strategies as well as multifaceted approaches, e.g. the combination of at least two strategies, were commonly used [9, 16]. It is not known which strategies were used to implement care bundles nor which ones are the most effective. Therefore, we conducted a systematic review to determine the strategies used to implement care bundles in adult ICU settings and to assess the effects of these strategies when implementing care bundles. We addressed the following questions: which strategies were used to implement the three most used care bundles, i.e. central line, ventilator and sepsis bundles, on adult ICUs and which implementation strategy or strategies lead to the highest levels of compliance?

## Methods

### Study design

A systematic review was conducted to determine the strategies used to implement care bundles in adult ICU settings and to assess the effects of these strategies when implementing care bundles. The protocol for the systematic review was not registered.

### Selection criteria

We included studies of any design which implemented one of the three mostly used care bundles, i.e. central line, ventilator or sepsis bundles, on ICUs for adult patients. Studies were only included if a description of the implementation strategy was given and if the level of compliance of the whole bundle or either compliance for each bundle element was reported separately. Studies written in non-English language were excluded. Protocols, abstracts, letters, commentaries or editorials were also not eligible.

### Search strategy

Systematic and comprehensive searches were developed with a clinical librarian and designed for optimal retrieval. The electronic databases MEDLINE/PubMed, Ovid/Embase, CINAHL and CENTRAL were searched for literature until January 31, 2015. The complete list of search terms and strategy of MEDLINE/PubMed can be found in Additional file 1. Additionally, the reference lists of included articles were checked.

### Inclusion of relevant studies

Two reviewers independently selected the studies (MB/DD or MB/AG). In case of discrepancies in study selections, we reached consensus through discussion. A third reviewer (DD or AG) was involved in case of disagreement. Studies were selected if they reported about the following: (1) central line, ventilator or sepsis bundle; (2) implementation strategies used; and if (3) compliance levels for the whole care bundle was reported or for each bundle intervention separately. Two criteria for selecting studies, i.e. compliance rates and implementation strategies, were not (clearly) reported in abstracts, while these criteria could be well described in the full-text. Therefore, if there was uncertainty whether a study reported about one of these two inclusion criteria, it was selected for full-text screening. Full-text articles were thoroughly reviewed, and studies were included if all three selection criteria were clearly described.

### Data extraction

Data extraction was performed by using a pre-defined data-abstraction sheet. The following data were extracted: author, publication year, research design, setting, participants, i.e. bundle users such as nurses or physicians, type of care bundle, implementation strategies, bundle elements, compliance rates and the type of compliance measurements. Two reviewers performed data extraction independently. In case of discrepancies, consensus was reached by discussion. A third reviewer was consulted in case consensus could not be reached.

### Quality assessment

A great variety exists in quality assessment tools for non-randomised studies. A valid checklist to assess the quality is currently lacking [17]. However, Downs and Black designed a checklist to evaluate the methodological quality of studies with both randomised and non-randomised designs [18]. We have used this tool to assess the risk of bias among the included studies. Checklist item number 27 about sample size calculation was simplified to a score of 0 (no sample size calculation) or 1 (sample size calculation reported). Therefore, a maximum score of 28 could be achieved for randomised studies and 25 for non-randomised studies. The following cut-off points have been reported to categorise studies by quality: excellent (26–28), good (20–25), fair (15–19) and poor (≤14) [19, 20]. Two reviewers conducted the quality assessment independently. Disagreement between the reviewers was resolved through discussion. A third reviewer was involved in case of disagreement.

### Implementation strategies

The different strategies that were used for implementation were categorised using the taxonomy developed by the Cochrane Effective Practice and Organisation of Care Group (EPOC) for dissemination and implementation strategies (Table 1) [21]. Where more than one method was used within one of the categories, this was measured as one strategy, i.e. if checklists and dashboards were used, this was categorised as a ‘reminder’ and was therefore measured as only one strategy.

**Table 1:** Explanation of the implementation strategies using the EPOC taxonomy [21–23]

Implementation strategy	Examples within the implementation of care bundles
Professional interventions	
Distribution of educational materials	(Web based) toolbox with educational materials, written material for self-study
Educational meetings	Educational meetings, seminars, workshops, teaching sessions
Local consensus processes	Development care bundle or materials or discussing about patients who developed an infection
Educational outreach visits	Use of a trained person who met professionals on the ICU to give information with the intent of changing practice
Local opinion leaders	Nursing and/or medical leadership
Audit and Feedback	Audits and feedback on infections rates or bundle compliance. Use of dash boards
Reminders	(Run) charts, checklists with bundle elements, daily goal sheets, insertion, HOB alarms
Tailored	Focus groups or (survey to) identify barriers
Mass media	Posters, fact sheets, newsletters, brochures to reach a great number of staff
Other; Time-out procedure	Time-out procedure, empower to stop procedure
Patient interventions	
Patient-family interventions	Family education of the bundle elements or family participation
Organisational interventions	
Revision of professional roles	Shifting of roles among staff
Clinical multidisciplinary teams	(Daily) multidisciplinary rounds, multidisciplinary teams
Skill mix changes	Changes in the number of staff
Continuity of care	Group of doctors to remove catheters daily
Satisfaction of providers	Nursing and medical champions, material rewards and staff engagement
Other; Implementation teams	Special team is actively involved to implement the care bundle, improvement teams
Structural interventions	
Changes in medical record system	Changes in a medical record system for electronic documentation

The EPOC taxonomy contains more items. We only used the taxonomy which was relevant in our study

### Types of measurements for care bundle compliance

Four different types of measurements were described in the literature to calculate the levels of bundle compliance: (1) ‘AON measurement’, which calculates the percentage of all indicated elements the patients actually have received, unless medically contraindicated [4, 24, 25]; (2) composite measurement, which can be calculated as a ratio between care that was actually given divided by the care that should have been given [24, 25]; (3) item-by-item measurement, which presents the nominator and denominator of each bundle element separately [25]; and (4) lowest level of compliance, which means that the lowest level of compliance to one of the elements is considered as the total bundle compliance [5, 7].

### Data analysis/synthesis

We used the compliance levels, which were last recorded as the measure of effect of implementation. Compliance was summarised as a percentage and, if applicable, as a numerator and a denominator. When studies were described as quality improvement initiatives, we further classified the nature of the study design by two reviewers independently. In case of discrepancies, consensus was achieved through discussion. We determined if selective reporting of compliance levels occurred within the included studies. Data analysis was performed in two phases. Firstly, overviews were given of all included studies to give insight in the study characteristics, compliance levels, the implementation strategies used, the number and types of bundles and their elements and the methods used to calculate compliance. In this phase, studies were not excluded based on their methodological quality. Secondly, a subgroup analysis was performed. For the subgroup analysis, the methodological quality of studies was assessed. In case a study scored less than 14 points, i.e. poor quality, it was excluded. Furthermore, subgroup analysis was not performed if less than three data points were available per subgroup. Studies were stratified and analysed by study design, quality assessment outcome, type of compliance measurement and by type of bundle. Subsequently, data were grouped and analysed by factors that could influence compliance, i.e. number of implementation strategies, bundle elements, methods for calculating compliance. From this, we attempted to identify patterns in compliance levels. Pearson’s product-moment correlation coefficient or Spearman’s rank order were used to assess the relationship of compliance to the number of implementation strategies and the relationship between compliance and the number of elements. Kendall’s rank correlation assessed the relationship of compliance to the time frame in which compliance was calculated. R (version: 3.1.3; R Foundation for Statistical Computing, Vienna, Austria) was used to perform subgroup analysis. Although a meta-analysis was planned, this could not be conducted due to the heterogeneity of the data in study designs, interventions and outcomes. Therefore, a narrative synthesis of the data is presented. This systematic review follows the standards of the preferred reporting items for systematic reviews and meta-analysis (PRISMA) [26].

## Results

**Fig. 1:**
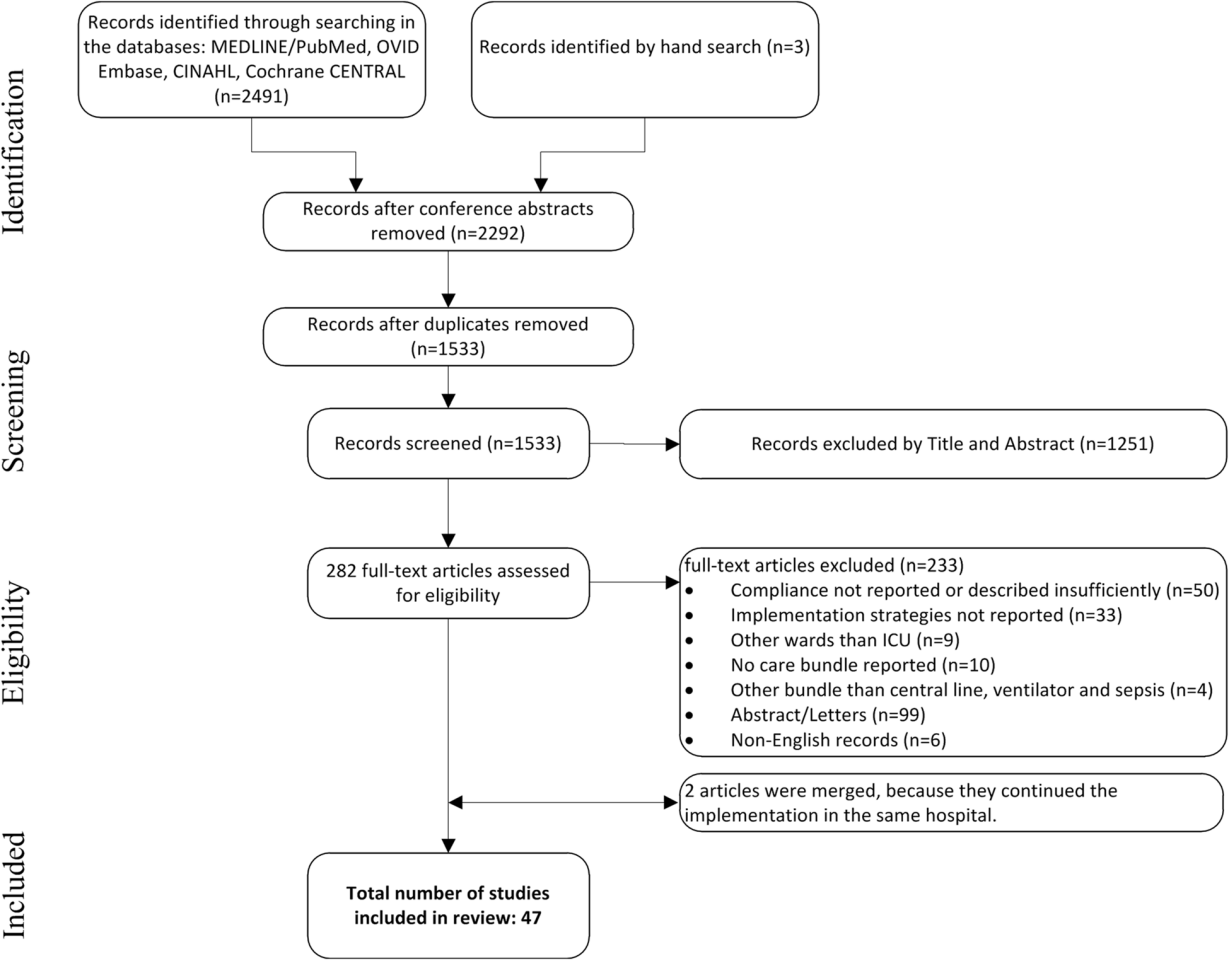
Flow chart of the study selection procedure

In total, 1533 records were identified for possible inclusion through the initial search, of which a final set of 47 studies met the inclusion criteria (Fig. 1).

### Quality assessment

Seventy-seven percent (36/47) of the studies scored between 15 and 19 points on the Downs and Black quality assessment scale and were classified as ‘fair’. Thirteen percent (6/47) of the studies scored 20 points or more and were classified as ‘good’. Eleven percent of the studies were classified as ‘poor’ (5/47) (Additional file 2). We assessed reporting bias of the included studies, and no studies were found reporting negative results.

### Study characteristics

Overall, 72 % (34/47) of the studies were conducted in a single hospital and 28 % (13/47) in two or more hospitals. The 47 studies that were included reported about the implementation of 49 care bundles. Thirteen studies described the implementation of the central line bundle [27–39], 27 studies described implementation of the ventilator bundle [10, 16, 35, 39–63] and nine studies described the sepsis bundle implementation [64–72] (Additional file 3). Two studies reported the implementation of two bundles, i.e. both central line and ventilator bundle [35, 39], and two studies were merged because they continued the implementation in the same hospital [60, 61]. One study [50] reported detailed information about the study participants, i.e. bundle users. They described variables as age, gender and years of work experience. The remaining studies only mentioned the type of disciplines that used the bundle without reporting additional information about the users. Studies about central line implementation used pre/post designs in 46 % (6/13), prospective cohort studies in 39 % (5/13) and retrospective designs in 15 % (2/13). Studies about the implementation of the ventilator bundle were conducted with pre/post designs in 48 % (13/27), with prospective cohorts in 33 % (9/27), as a longitudinal study in 4 % (1/27) and as both interrupted time series and retrospective designs in 7 % (2/27). For the studies about sepsis bundle implementation, pre/post designs were used in 56 % (5/9) and prospective cohort designs in 44 % (4/9). A detailed description of relevant study characteristics is shown in Additional file 3, which is organised by type of bundle and study design.

### Number of care bundle elements

Both the number of elements per bundle and the types of element varied (Additional file 4). Three types of central line bundles were described: (1) central line bundle in general (*n* = 8), (2) insertion bundle (*n* = 5) and (3) maintenance bundle (*n* = 3). The range of elements within the central line bundle varied from three to seven elements (Additional file 4). In 8/16 central line bundles, five elements were included and most of these elements were derived from the original IHI bundle [5]. The number of elements per ventilator bundle ranged from four to seven. In 12 studies (44 %, 12/27), the bundle consisted of four elements, and in three studies [50, 58, 62] (11 %, 3/27), the bundle contained seven elements (Additional file 4). The most common element was ‘elevation of the head-of-the-bed’ in 96 % (26/27), followed by deep venous thrombosis prophylaxis and peptic ulcer prophylaxis in 78 % (21/27). The sepsis bundle was divided into the resuscitation bundle (*n* = 5) and management bundle (*n* = 6). In two studies [67, 68], the general sepsis bundle contained six and 11 elements, respectively. The resuscitation bundle has a range of five to seven elements, while the management bundle contains two to four elements (Additional file 4).

### Implementation strategies

The three most frequently used strategies to implement care bundles were as follows: educational activities in 88 % (43/49) followed by reminders in 71 % (35/49) and audit and feedback (A&F) in 63 % (31/49). Family participation was only adopted as a strategy to implement the ventilator bundle (Table 2). Within each study about central line implementation, a minimum of one strategy was described, ranging from one to a maximum of seven strategies. In all studies of central line bundle implementation, checklists were used. Education was used in 85 % (11/13) and A&F in 77 % (10/13). In 54 % (7/13), the time-out procedure was reported (Additional file 3). In studies of the implementation of the ventilator bundle, there is a great variety in the number of strategies, ranging from one to nine strategies (Table 2). The three most frequently used strategies were education (85 %, 23/27), reminders (78 %, 21/27) and A&F (67 %, 18/27). In studies of the implementation of the sepsis bundle, education was most frequently used (89 %, 8/9), followed by mass media strategies, e.g. distribution of posters (44 %, 4/9). In contrast with the strategies to implement the central line and ventilator bundles, the concept of a reminder was only used in one study of the implementation of the sepsis bundle [66] (Table 2).

**Table 2:** Implementation strategies

	Central line bundle	Ventilator bundle	Sepsis bundle	Total number
Professional interventions				
Distribution of educational materials	27, 32–34	10, 40, 41, 46, 52, 56	66–70, 72	16
Educational meetings	28, 30, 35, 36	35, 41, 42, 53, 59	66, 67, 69, 70, 72	14
Local consensus processes		45, 46, 51, 57		4
Educational outreach visits	27–29, 31–34, 36, 37	10, 40, 42–60/61, 63	64–66, 68	34
Local opinion leaders	34, 36		65	3
Audit and Feedback	27, 28, 30–34, 36–38	10, 16, 40, 41, 43, 44, 46, 49, 52–54, 56–62	65, 66, 70	30
Reminders	27–39	10, 35, 39–47, 49, 51–54, 56–59, 63	65	35
Tailored		41, 51, 53, 54, 59		5
Mass media	27, 28, 30, 32	10, 40, 44, 45, 47, 52, 53, 56, 57, 59–62	65–67, 72	20
Other; Time-out procedure	28–30, 34, 36, 38	49, 54, 60/61		9
Patient interventions				
Patient-family interventions		46, 57, 59		3
Organisational interventions				
Revision of professional roles		59		1
Clinical multidisciplinary teams	28, 35	10, 35, 41, 43, 53, 55, 56, 57, 59, 63	68	13
Skill mix changes			68, 69, 71	3
Continuity of care	30			1
Satisfaction of providers	31, 33, 36	40, 46, 48, 54, 56		8
Other; Implementation teams	27, 29, 31, 34–36	35, 42, 45, 46, 52, 53, 56	65, 68, 69	16
Structural interventions				
Changes in medical record system	38		64	2

The numbers in the table are reference numbers, except for those in the last column

Central line bundle: 13 studies; Ventilator bundle: 27 studies; Sepsis bundle: 9 studies

### Type of compliance measurements

In the majority of the studies, the AON approach was used (*n* = 36). The composite measurement was reported in four studies [33, 46, 53, 63]. Three studies [38, 39, 52] reported the lowest level of compliance, two studies [45, 72] used the item-by-item measurement (Additional file 3) and in two studies the type of measurement was not clearly reported. In nine studies on the central line bundle implementation, the AON approach was reported to calculate the compliance levels. In two studies, the composite measurement was used and in one study, the lowest level of compliance. In one study, the type of measurement could not be identified. Exline et al. reported a high level of compliance of 100 % with the insertion bundle, using the AON approach [36]. In the study of Render et al., the compliance with the central line bundle was 98 % at the end of the study period using the composite measurement [33]. One study [27] reported a low compliance rate of 44 %, which was measured over a period of 18 months (Additional file 3). In the calculation of the compliance of the ventilator bundle, four types of measurements were used. One study reported the compliance per single item [46], three studies used the composite measurement and two studies used the lowest level of compliance. In the remaining studies, the AON approach was used to measure the compliance of the ventilator bundle (Additional file 3).

### Time frame compliance calculation

Compliance was calculated over different time frames, i.e. some studies calculated compliance for each month while others measured the overall compliance over a longer period, i.e. 1 or 2 years. In three studies about ventilator bundle implementation [57, 59–61], compliance rates of 100 % were reached. In these studies, the compliance was calculated monthly by using the AON approach. Two studies reported low compliance levels of 30 and 34 %, respectively [42, 52]. In these studies, the compliance was measured using the AON approach over the whole study period (Additional file 3). In most studies about sepsis bundle implementation, the level of compliance was measured using the AON approach. Only one study [66] used the item-by-item measurement to report compliance. The compliance levels for sepsis bundles were exceptionally low compared to the central line and ventilator bundles (Additional file 3). Two studies reported compliance levels of 68 % [64] and 70 % [68], respectively. However, these studies were performed in small patient numbers.

### Effects on compliance

The first subset of studies that was analysed included studies with pre/post designs, which were qualified as either good or fair and in which compliance was calculated by using the AON approach. Additional file 5: Figure S1 shows that, overall, there is no association between the number of strategies used and compliance levels (*r* = 0.118, 95 % CI −0.331 to 0.523, *p* = 0.612). The same applies when the bundles are analysed separately. As shown in Table 2, different strategies were used in combination for implementation of care bundles. For the implementation of the central line and ventilator bundle, the combination of education, reminders and A&F was used. For the implementation of the sepsis bundle, education is mainly used in combination with distribution of educational materials. Overall, there is an association neither between compliance and the number of elements (*ρ* = 0.140, *p* = 0.545) nor between compliance and the time frame used to calculate compliance (*τ* = −0.080, *p =* 0.639). The second subset of studies that was analysed included prospective cohort studies with quality assessments of either good or fair and in which compliance was calculated using the AON approach. Additional file 5: Figures S4 to S6 show that there is a variety in compliance levels. Moreover, no association can be found between the number of implementation strategies (*ρ* = 0.539, *p* = 0.057), bundle elements (*ρ* = −0.303, *p* = 0.314) and time frame used for measuring compliance (*τ* = −0.189, *p* = 0.417).

## Discussion

In this systematic review, we identified the strategies that were reported to implement care bundles in ICU settings, and subsequently, we attempted to find the best strategies to achieve high levels of bundle compliance. Care bundles have already proven to be effective in reducing negative clinical outcomes [7, 9, 10]. This reduction is associated with the compliance rates to the care bundles [12]. It is important to mention that we, therefore, focused on finding the best implementation strategy to achieve high levels of bundle compliance and not on the outcome of care processes. Although care bundles are perceived as valuable and are proven to have an effect on the quality of care, it is still a challenge to achieve high levels of bundle compliance.

Our results show that the three most frequently used implementation strategies were education followed by reminders and A&F. These findings are consistent with other reviews about implementation strategies in general [73, 74], in which these three strategies were commonly used to implement best practices in hospitals [73] or critical care areas [74]. In 53 % of the studies, a combined strategy consisting of education, reminders and A&F were used. This combination was mainly used to implement the ventilator bundle (57 %), and only used in 11 % for implementing the sepsis bundle. Overall, after implementation of the bundles, compliance levels varied, ranging from 33 to 100 %. However, these findings should be interpreted with caution, because studies included in this systematic review showed a variety of designs. The majority of studies involved quality improvement initiatives with pre/post designs or prospective cohort studies without using controls. For these studies, secular trends that might have occurred at the same time were not taken into account. Furthermore, we assessed the quality of the individual studies by using the checklist of Downs and Black [18] and the majority of the studies were classified as fair. Remarkably, none of the studies provided more detailed information about the participants, i.e. bundle users, except for one [50]. Information about the setting was reported in all studies. Such details about the context of an intervention should be reported to determine the generalizability, or external validity, of the study [75, 76]. We furthermore determined great differences in the number and types of bundle elements between the studies, and in the measurements and calculations of bundle compliance rates. Due to this heterogeneity of data, even within the different subgroups (Additional file 5), we could not identify the most effective implementation strategy that resulted in the highest levels of compliance. In the next paragraphs, we will discuss how these factors could have influenced the compliance levels.

### Number of elements per bundle

The total number of elements per bundle varied, with a range of three elements in the central line bundle [36] to 11 in the sepsis bundle (Additional file 4) [68]. The concept of a care bundle is to have a small number of elements to ensure that evidence-based care will be delivered reliably [4]. Adding more elements is likely to affect the reliability of the bundle, i.e. if more elements are included, it is more difficult to perform all bundle elements at once. Consequently, this results in lower compliance levels [4].

### Differences in types of bundle elements

Our results show that even within one group of bundles, different types of elements were added. Hospitals design their own care bundle and when including elements, it is important that each element is generally accepted by hospital staff [4, 8]. The reliability of these new elements, as well as the acceptance of an element (intervention), may affect the likelihood and motivation to use the bundle [3, 4]. One study compared the compliance rates of three different sepsis bundles. In this comparative study, several factors were observed which were affecting the compliance rates, such as the exclusion criteria for an intervention and the definition of an intervention [77].

### Time period compliance calculation

Our results show that four different types of measurements were used to calculate the compliance levels. In most studies, detailed information about compliance rates was not reported at all. In most studies, the AON approach was used [4–6], and therefore, it is possible that lower compliance levels were reported. Compared to the AON approach, the composite measurement has greater sensitivity for giving insight in the changes in care processes [24, 25]. Benneyan recommends both measurements because of their specific benefits [24]. In some studies, the bundle compliance was measured monthly, while other studies measured compliance over a longer period of time, i.e. over a period of several months or years. In most studies, detailed information about compliance, such as the monthly numerators and denominators, was not reported.

Among the included studies, the success of bundle implementation was highly variable, even when studies were stratified on design, methodological quality and type of measurement. This could be explained by either the number and types of bundle elements or by the ways compliance is measured and calculated as shown in this systematic review. Differences in measuring and reporting performance outcomes were observed by Dixon-Woods et al. [78]. In their analysis of a national programme to reduce the rates of central venous catheter bloodstream infections, they found that the standardised definitions and measurements of the study outcomes were interpreted differently between the participating ICUs. This resulted in differences in collecting data, and therefore, data between ICUs were not fully comparable [78].

The variety in compliance rates could be influenced by other factors. Bundle compliance is often monitored by using checklists [79] (Additional file 3). Besides auditing compliance, checklists are useful tools to standardise care processes, comparable to care bundles, and to improve the reliability of care to ensure patients receive all evidence-based interventions needed [79]. Although the use of checklists is promising, it is known that they are underused and barriers exist to use them which negatively influence the reliability of care [79, 80]. Thus, there could be a discrepancy between actual delivered care and the use of checklists, resulting in lower compliance rates, while the care was actually performed. Another example is that compliance of a new intervention could be negatively influenced when related to the habits and positive beliefs regarding the ‘old’ intervention even when the new intervention is based on robust science [27]. Furthermore, one study showed that lack of monitoring compliance was the reason for non-compliance [50]. Complementary, the frequency of monitoring compliance has resulted in positive effects on bundle compliance rates [81]. Monitoring data, e.g. on compliance and/or infection rates, results in increased awareness and encourages ICU staff to be compliant with the care bundle.

Although desirable, it can be challenging to achieve and maintain levels of bundle compliance of more than 95 % [4, 9]. In order to sustain the success of implementation, change of the organisational culture into a safety culture is required [9, 82]. Creating a culture of safety includes the change of behaviour or attitudes of hospital staff to openly discuss about patient safety-related issues and to learn from mistakes without blaming [13]. Creating a culture of safety is necessary to enhance the adoption of care bundles, which subsequently contributes to redesign care processes and improve team work and communication between professionals [4, 9].

Implementation of quality improvement projects does not have to give the same positive findings when reproduced in other hospitals. One example is the Keystone project in Michigan which showed a sharp decline in the central line infection rates in ICUs [9]. Many of the components of this project were replicated in ICUs in the UK which also showed a reduction in infection rates. However, these positive findings were not only due to the multifaceted interventions of the programme used but were part of a secular trend. Secular trends are not often measured in quality improvements [83, 84], i.e. studies about implementing quality initiatives are often part of larger hospital or nationwide improvement programmes which positively influences patient outcomes as well. The context in which a programme for quality improvement is launched contributes to different outcomes [83, 85].

### Limitations

Our systematic review is hampered by several limitations. There is a chance that we missed some relevant studies, because different terms are given to care bundles. However, a broad search strategy was used and we have completed the search with a hand search. Two criteria for selecting studies, i.e. compliance rates and implementation strategies, were not (clearly) reported in abstracts, while these criteria were described in the full text. We included any article to the phase of full-text screening if there was any uncertainty about one of the inclusion criteria. Furthermore, our review was restricted to the inclusion of English language publications only and relevant studies published in other languages could have been missed. However, evidence for the effect of language restrictions on systematic bias remains inconclusive. Another important issue is that no studies with randomised designs were included. The majority of the studies included were quality improvements and before-and-after studies without controls. Thus, observed changes could be influenced by secular trends [86]. Furthermore, the overall methodology of the included studies was poor, involving an increased risk of bias [86]. Therefore, the results should be interpreted with caution. An important problem hampering a meta-analysis was due to the heterogeneity of the available data (Additional file 5). There was a high variability in study design, methodological quality, bundle characteristics, compliance measurements and the calculation of compliance within a specific time frame. Therefore, it was not possible to point out the superior implementation strategy. Moreover, complete data of compliance was lacking, e.g. most studies only reported compliance as a percentage, without explicitly reporting numerators and denominators. Although not all included studies show high compliance levels, publication bias could still have influenced our results since all included studies show positive results. Since compliance was reported as secondary outcome, the quality of reporting could have been influenced by this fact.

### Future research

Further research is needed to identify the best strategy to implement care bundles to achieve high levels of compliance. To investigate the effects of implementation strategies on compliance levels, there is a need for robust study methods in implementation or quality improvement research. Studies using randomised designs should be considered to increase the internal and external validity, especially when the intervention is considered for widespread implementation [87]. However, randomization is not always possible or suitable in quality improvement studies. Alternative designs could then be considered, such as controlled before-and-after trials or interrupted time series to control for confounding variables [88]. Otherwise, a combination of quantitative and qualitative designs could be conducted to assess if the intervention worked, how it worked and in what contexts [83, 88]. Furthermore, it is imperative that studies are clearly and unambiguously reported. A clear description about the context in which the intervention was implemented should be stated, and a detailed description of the participants, i.e. the users of the intervention, should be provided [75]. These requirements are stipulated in the standards for quality improvement reporting excellence (SQUIRE) guidelines [75] which are strongly recommended when reporting quality improvement studies. To compare performance outcomes, there should be an unambiguous method for measuring compliance, i.e. the use of the AON and/or composite measurement [24]. Within current implementation research, it is not only important to identify the most effective strategy, but also to better understand why, how and when the specific strategy works best [89].

## Conclusions

The three most frequently used implementation strategies were education, reminders and audit and feedback. We conclude that the heterogeneity among the included studies was high due to the variety in study design, difference in number and types of elements, types of compliance measurements calculation. Due to the heterogeneity of the data and the poor methodological quality of the studies, conclusions about which strategy results in the highest levels of care bundle compliance could not be determined and no recommendations can be made on which strategy should be selected to get the highest levels of compliance. We strongly recommend that studies in quality improvement should be reported in a formalised way in order to be able to compare research findings. It is imperative that authors follow the SQUIRE guidelines whenever they report quality improvement studies.

## Additional files

Additional file 1:
**Search strategy.** (PDF 17.5 KB) Additional file 2:
**Quality assessments.** (PDF 41.3 KB) Additional file 3:
**Study characteristics in detail.** (PDF 87.2 KB) Additional file 4:
**Bundle elements.** (PDF 150 KB) Additional file 5:
**Effects on compliance.** (PDF 115 KB)

## Abbreviations

A&F: audit and feedback
AON: all-or-none
EPOC: effective practice and organisation of care group of the Cochrane centre
ICU: intensive care units
IHI: Institute for Healthcare Improvement
SQUIRE: standards for quality improvement reporting excellence
