# Supplementary material for: What are effective strategies for the implementation of care bundles on ICUs: a systematic review
Source: Implement Sci. 2015 Aug 15;10:119. doi: 10.1186/s13012-015-0306-1 (PMC4536788; doi:10.1186/s13012-015-0306-1)
Supplement: Additional file 1: — Search strategy. (PDF 17.5 KB) [file 13012_2015_306_MOESM1_ESM.pdf]

Additional file 1 Search strategy MEDLINE/PubMed

(("Intensive Care Units"[Mesh] OR Intensive Care*[tiab] OR ICU*[tiab] OR critical care*[tiab] OR "Critical Care"[Mesh]) AND (bundle*[tiab] OR evidence based*[tiab] OR "evidence-based practice"[MeSH]) AND (ventilat*[tiab] OR pneumon*[tiab] OR sepsis[tiab] OR VAP[tiab] OR CRBI*[tiab] OR CLABSI*[tiab] OR CVL*[tiab] OR central line*[tiab] OR bloodstream*[tiab] OR "Pneumonia, Ventilator-Associated"[Mesh] OR "Catheter-Related Infections"[Mesh] OR sepsis[Mesh]) AND ("Guideline Adherence"[Mesh] OR compliance[tiab] OR adherence[tiab] OR guideline*[tiab] OR implement*[tiab] OR improve*[tiab] OR disseminat*[tiab] OR intervent*[tiab] OR mail*[tiab] OR educat*[tiab] OR leader*[tiab] OR remind*[tiab] OR didac*[tiab] OR multifaceted*[tiab] OR strateg*[tiab] OR tailored interv*[tiab] OR feedback*[tiab] OR audit*[tiab]))
