# Supplementary material for: What are effective strategies for the implementation of care bundles on ICUs: a systematic review
Source: Implement Sci. 2015 Aug 15;10:119. doi: 10.1186/s13012-015-0306-1 (PMC4536788; doi:10.1186/s13012-015-0306-1)
Supplement: Additional file 2: — Quality assessments. (PDF 41.3 KB) [file 13012_2015_306_MOESM2_ESM.pdf]

Additional file 2 Quality assessments using the 27 criteria of the checklist of Downs & Black [18]

**Central line bundle**	**1**	**2**	**3**	**4**	**5**	**6**	**7**	**8**	**9**	**10**	**11**	**12**	**13**	**14**	**15**	**16**	**17**	**18**	**19**	**20**	**21**	**22**	**23**	**24**	**25**	**26**	**27**	**Total score**
Marra, 2010	1	1	1	1	0	1	1	0	1	1	1	1	1	0	0	1	1	1	1	1	1	1	0	0	0	1	0	19
Jeong, 2013	1	1	1	1	1	1	1	0	1	1	1	1	1	0	0	1	1	1	1	1	1	1	0	0	0	1	0	20
Richardson, 2012	1	1	0	1	0	0	0	0	1	0	1	1	1	0	0	0	1	0	1	0	1	1	0	0	0	0	0	11
Bonello, 2008	1	1	0	1	1	0	0	0	1	0	1	0	1	0	0	1	1	0	1	1	1	1	0	0	0	1	0	14
Exline, 2013	1	1	0	1	1	1	1	0	1	1	1	1	1	0	0	1	1	1	1	1	1	1	0	0	0	1	0	19
Longmate, 2011	1	1	1	1	0	1	1	0	1	1	1	1	1	0	0	1	1	1	1	1	1	1	0	0	0	1	0	19
McPeake, 2012	1	0	0	1	0	0	0	0	1	0	1	0	1	0	0	1	1	0	1	1	1	1	0	0	0	1	0	12
Hocking, 2013	1	1	1	1	0	1	1	0	1	1	1	0	1	0	0	1	1	1	1	1	1	1	0	0	0	1	0	18
Render, 2011	1	1	0	1	0	1	1	0	1	1	1	1	1	0	0	1	1	1	1	1	1	1	0	0	0	1	0	18
McNamara, 2011	1	1	0	1	0	1	0	0	1	0	1	1	1	0	0	1	1	0	1	1	1	1	0	0	0	1	0	15
Helmick, 2014	1	1	0	1	0	0	1	0	1	1	1	0	1	0	0	1	1	1	1	1	1	1	0	0	0	1	0	16
Khalid, 2013	1	1	0	1	0	0	1	0	1	1	1	0	1	0	0	1	1	1	1	1	1	1	0	0	0	1	0	16
Sacks, 2014	1	1	1	1	0	1	1	0	1	1	1	1	1	0	0	1	1	1	1	1	1	1	0	0	0	1	0	19

**Ventilator bundle**	**1**	**2**	**3**	**4**	**5**	**6**	**7**	**8**	**9**	**10**	**11**	**12**	**13**	**14**	**15**	**16**	**17**	**18**	**19**	**20**	**21**	**22**	**23**	**24**	**25**	**26**	**27**	**Total score**
Hatler, 2006	1	0	1	1	0	0	0	0	1	0	1	1	1	0	0	1	1	0	1	1	1	1	0	0	0	1	0	14
Malouf T,2013	1	1	0	1	0	1	1	0	1	1	1	1	1	0	0	1	1	1	1	1	1	1	0	0	0	1	0	18
Lawrence, 2012	1	1	0	1	0	1	1	0	1	1	1	1	1	0	0	1	1	1	1	1	1	1	0	0	0	1	0	18
Morris, 2011	1	1	1	1	0	1	1	0	1	1	1	1	1	0	0	1	1	1	1	1	1	1	0	0	0	1	0	19
Hawe, 2009	1	1	1	1	1	1	1	0	1	1	1	1	1	0	0	1	1	1	1	1	1	1	0	0	0	1	0	20
Bloos, 2009	1	1	1	1	0	1	1	0	1	1	1	1	1	0	0	1	1	1	1	1	1	1	0	0	0	1	0	19
Rello, 2013	1	1	1	1	0	1	1	0	1	1	1	1	1	0	0	1	1	1	1	1	1	1	0	0	0	1	0	19
Bukhari, 2012	1	1	1	1	0	0	0	0	1	1	1	1	1	0	0	1	1	0	1	1	1	1	0	0	0	1	0	16
Marra, 2009	1	1	1	1	1	1	1	0	1	1	1	1	1	0	0	1	1	1	1	1	1	1	0	0	0	1	0	20
Caserta, 2012	1	1	1	1	1	1	1	0	1	1	1	1	1	0	0	1	1	1	1	1	1	1	0	0	0	1	0	20
Esmail, 2008	1	1	1	1	0	1	0	0	1	0	1	1	1	0	0	0	1	0	1	1	1	1	0	0	0	1	0	15
Youngquist, 2007	1	0	0	1	0	1	0	0	1	1	1	1	1	0	0	1	1	0	1	1	1	1	0	0	0	1	0	15
Zaydfudim, 2009	1	1	1	1	0	1	1	0	1	1	1	1	1	0	0	1	1	1	1	1	1	1	0	0	0	1	0	19
Al-Tawfiq, 2010	1	1	1	1	0	1	0	0	1	0	1	1	1	0	0	1	1	1	1	1	1	1	0	0	0	1	0	17
Bonello, 2008	1	1	0	1	1	0	0	0	1	0	1	0	1	0	0	1	1	0	1	1	1	1	0	0	0	1	0	14
Miller, 2010	1	1	1	1	0	1	1	0	1	1	1	1	1	0	0	1	1	1	1	1	1	1	0	0	0	1	0	19
DePalo, 2010	1	1	0	1	0	1	1	0	1	1	1	1	1	0	0	1	1	1	1	1	1	1	0	0	0	1	0	18
Jimenez, 2009	1	1	1	1	0	0	1	0	1	1	1	1	1	0	0	1	1	1	1	1	1	1	0	0	0	1	0	18
DuBose, 2008	1	1	0	1	0	0	1	0	1	0	1	1	1	0	0	0	1	0	1	1	0	0	0	0	0	1	0	12
Berenholtz, 2011	1	1	1	1	1	1	1	0	1	1	1	1	1	0	0	1	1	1	1	1	1	1	0	0	0	1	0	20
Crunden, 2005	1	1	1	1	0	1	1	0	1	0	1	1	1	0	0	1	1	1	1	1	1	1	0	0	0	1	0	18
Berenholtz, 2004	1	1	0	1	1	1	1	0	1	1	1	1	1	0	0	1	1	1	1	1	1	1	0	0	0	1	0	19
Eom, 2014	1	1	1	1	0	0	1	0	1	0	1	1	1	0	0	1	1	1	1	1	1	1	0	0	0	1	0	17
Hamishehkar, 2014	1	1	1	1	0	1	1	0	1	1	1	1	1	0	0	1	1	1	0	0	1	1	0	0	0	1	0	17
Helmick, 2014	1	1	0	1	0	0	1	0	1	1	1	0	1	0	0	1	1	1	1	1	1	1	0	0	0	1	0	16
Lim, 2013	1	1	1	1	0	1	1	0	1	1	1	1	1	0	0	1	1	1	1	1	1	1	0	0	0	1	0	19
Mukhtar, 2014	1	1	1	1	0	1	1	0	1	1	1	1	1	0	0	1	1	1	1	1	1	1	0	0	0	1	0	19
Al-Thaqafy, 2014	1	1	1	1	0	1	1	0	1	1	1	1	1	0	0	1	1	1	1	1	1	1	0	0	0	1	0	19

**Sepsis bundle**	**1**	**2**	**3**	**4**	**5**	**6**	**7**	**8**	**9**	**10**	**11**	**12**	**13**	**14**	**15**	**16**	**17**	**18**	**19**	**20**	**21**	**22**	**23**	**24**	**25**	**26**	**27**	**Total score**
Memon, 2012	1	1	1	1	0	1	1	0	1	1	1	1	1	0	0	0	1	1	1	1	1	1	0	0	0	1	1	19
Schramm, 2011	1	1	1	1	0	1	1	0	1	1	1	1	1	0	0	1	1	1	1	1	1	1	0	0	0	1	0	19
Kim, 2012	1	1	1	1	0	1	1	0	1	1	1	1	1	0	0	1	1	1	1	1	1	1	0	0	0	1	0	19
Guilliano, 2011	1	1	1	1	0	1	1	0	1	1	1	1	1	0	0	1	1	1	1	1	1	1	0	0	0	1	0	19
Ferrer, 2008	1	1	1	1	0	1	1	0	1	1	1	1	1	0	0	1	1	1	1	1	1	1	0	0	0	1	1	20
Laguna Perez, 2012	1	1	1	1	0	1	1	0	1	1	1	1	1	0	0	1	1	1	1	1	1	1	0	0	0	1	0	19
Castellanos, 2010	1	1	1	1	0	1	1	0	1	1	1	1	1	0	0	1	1	1	1	1	1	1	0	0	0	1	0	19
Lefrant, 2010	1	1	1	1	0	1	1	0	1	1	1	1	1	0	0	1	1	1	1	1	1	1	0	0	0	1	1	20
Silverman, 2011	1	1	1	1	0	1	1	0	1	1	1	1	1	0	0	0	1	1	0	1	1	1	0	0	0	1	0	17
