# Supplementary material for: What are effective strategies for the implementation of care bundles on ICUs: a systematic review
Source: Implement Sci. 2015 Aug 15;10:119. doi: 10.1186/s13012-015-0306-1 (PMC4536788; doi:10.1186/s13012-015-0306-1)
Supplement: Additional file 3: — Study characteristics in detail. (PDF 87.2 KB) [file 13012_2015_306_MOESM3_ESM.pdf]

Additional file 3 Study characteristics in detail

First author, Publication year, country, care bundle	Design	Study Periods	ICU (number of beds)	Study outcomes	Participants (bundle users)	Model of theory/ program of patient safety	Type of compliance measurement/ Period in which compliance is calculated	Implementation details	Effects on compliance
Jeong[27] 2013, South Korea, Central line bundle	pre/post design	Baseline: Apr'09- mrt '10, Intervention phase: Apr'10- Dec'11 - - Transition period: Apr-Sep'10 - Follow up: Oct '10-Dec'11	ICU (39 beds)	Bundle compliance. <br>Incidence of CLABSI. <br>Length of time between insertion and CLABSI occurrence	Health care professionals: nurses and physicians	NS	AON/ Overall: 18 months	1) Development Task Force team, 2) posters 3) distribution of educational programs and materials 4) feedback on unit level compliance with CL bundle and CLABSI incidence. 5) checklist. During follow up period (Oct '10-Dec '11): weekly feedback on compliance and monthly feedback on CLABSI rates. Task force team provided feedback on the overall follow up process. Educational programs and distributed materials to new professionals.	Phase 1: Baseline: 0% <br>Phase 2: Intervention period: 44,3% (p<0.001)
Hocking[28] 2013, New Zealand, Central line bundle	pre/post design	Baseline: Oct '07- Dec '08 Post intervention: Jan '09 - Apr '11.	General ICU (7 beds. By July '08: 12 beds)	CLABSI rate. <br>Bundle compliance.	Nurses and doctors. Nurse-pt ratio: 1:1 or 1:2	NS	AON/ Per month	1) education, 2) reminders during nurses shift handover and placed on computer screen, 3) checklist, 4) nurses observed insertion and they reminded doctors to comply with the bundle, 5) feedback on compliance, 6) posters, 7) teaching at the bedside in small groups. 8) face to face teaching sessions 9) Signs (updated each day) with run charts of CLAB rate and staff compliance, 10) daily rounds, 11) visual updates on staff compliance	Insertion bundle Dec'08: 36% to Apr '11: 81%. Maintenance bundle Jun '09: 76% to Apr '11: 80%. Compliance High risk bundle was not described
Sacks[29] 2014, USA, Central line bundle	Pre/post design	Baseline: Nov'04- Nov'05. Intervention: Dec'05-Mar'06. Post intervention	Tertiary care hospital, surgical ICU: 16 beds	CLABSI rates	Physicians and nurses	NS	AON/ Per month	1) multidisciplinary implementation team; 2) computerized training module and examination for all physicians and nurses involved; 3) nurses were instructed to stop the procedure if they noticed any violation; 4) checklist; 5) mobile central line insertion cart containing all equipment and supplies for insertion and management of CVCs	Overall compliance: March '06: 58% (19/33)
Marra[30] 2010, Brazil, Central line bundle	Quasi-experimental: pre/post design	Phase 1: Mrt'05- Mrt'07. Phase 2 (bundle implementation): Apr'07- Apr'09	Medical and surgical ICU (38 beds)	CLABSI rate	ICU-team: doctors and nurses	NS	AON/ Overall:2 years	1) Educational meetings, 2) monthly feedback on compliance via email. 3) Posters with bar charts displaying compliance + CLABSI rates. 4) Group of ICU doctors to remove catheters daily. 5) nurses intervened in the process at the same time if non-compliance with an element was detected, 6) insertion chart	Phase 2 on ICU: 1723/1833 (94%)
Longmate[31] 2011, UK, Central line bundle	Quality improvement: pre/post design	Baseline: 1 Sep'05- 31 Aug'06. Intervention: Mrt'08-Aug'09	Medical and surgical ICU, (9 beds)	CLABSI rates. <br>Care processes: reliability.	Nurses and doctors	Scottish Patient Safety program.	AON/ Per month	1) Active engagement of staff, 2) educational programs, 3) measurement and feedback of outcomes, 4) insertion checklist, 5) organizational change, 6) introduction of the Scottish Patient Safety program, 7) measurement of insertion processes and feedback, 8) ownership of nurses of the two bundles	Insertion bundle compliance per month: Mar '08: 80% to Aug '09: 95%
Khalid[32] 2013, Saudi Arabia,	Quality improvement: pre/post	Pre intervention: Febr '09-Jan '10 Postintervention	Tertiary care hospital, medical/surgi	CLABSI rates	Physicians and 101 nurses.	NS	Not clear/ Per month	1) education sessions; 2) audits, 3) checklists; 4) daily reminders; 5) flyers	Insertion bundle: Pre-int: 85% Postint yr 1: 96%,

Central line bundle	design	yr1: Aug'10-Jul'11<br>Yr 2: Aug'11-Jul'12	cal ICU: 18 beds		Nurse-pt ratio: 1:1, 1:2				yr 2: 99%.<br>Maintenance bundle: Pre intervention: 75%. Post int yr. 1: 91%, yr 2: 97%.
Render[33] 2011, USA, Central line bundle	Observational cohort study	Apr'06-Apr'09	Multicenter, General, medical, surgical and cardiac ICUs: 174 ICUs (1774 beds)	1. Adherence bundle elements<br>2. CLABSI rates	Nurses	PDSA cycles	Composite/ Per quarter	1) hospital leadership, 2) learning module, 3) physician champion, 4) use of central line cart, 5) checklist during line insertion as a forcing function, 6) addition of a daily goal sheet during physician rounds as a memory aid for CL removal. 7) feedback about CLABSI rate and bundle adherence 8) web based toolbox with education materials (for sharing in multicenter setting). 9) ICU dashboard progress in CLABSI rates in own ICU compared with national rates.	Apr to Dec '06: 85%, 2009: 98%
Richardson [34] 2012, USA, Central line bundle	Evidence Based Practice project/quality improvement: Prosp cohort	May '06- May '10	Medical and surgical ICU (Number of beds NS)	Infection rates	Nurses and physicians	NS	AON/ Per quarter	1) Implementation team of clinical nurse specialists, 2) education every 6 months (didactic lectures simulation laboratory, emails, quiz format etc), 3) checklist en modified this when needed, 4) nurses empowered to stop the procedure if any of the required bundle item practices were not being followed, 5) nursing and medical leadership, 6) 'Hot team' facilitated the group in determining the logistics for the work that needed to be done, they met monthly, established a web page on intranet and posted literature, relevant policy, procedure statements, education files as pp presentations, auditing forms, 7) feedback on compliance and infection rates.	Compliance during the whole study period: >90%.
Bonello[35] 2008, USA, Central line bundle	Quality improvement: Prosp cohort	Jan'05 - Jun'06	Multicentre, 12 ICUs, (95 beds)	Process measures: adherence to CL insertion checklist.<br><br>CRBSI rates.	ICU care providers	Quality Improvement Collaborative using the PDSA cycle	AON/ Per quarter	1) Educational symposia, 2) interdisciplinary team rounds, 3) checklist at bedside. 4) multidisciplinary implementation teams	CL bundle: First 3 months: 58%, Final 3 months: 74%
Exline[36] 2013, USA, Central line bundle	Observational cohort study with historical controls. Historic controls not used for compliance	Baseline: Jan'08-Dec'09. Intervention year 1: Jan-Dec'10, Intervention year 2: Jan-Dec'11	Medical ICU, (25 beds)	Quarterly CLABSI rates. Compliance with CVC insertion and dressing maintenance practices.	ICU nurses and physicians	NS	AON/ Audit per month	1) Insertion checklist, 2) Audit and feedback by infection preventionists: compliance per month and CLABSI per week, 3) Educational meetings, 4) Mandatory demonstration session for dressing change and proper line access on manikin, 5) Nurses were empowered to stop the procedure if sterile technique was not correctly employed. 6) Insertion trays were augmented with components to comply with central line bundle, 7) Coat pins 8) implementation teams, 9) unit physician and nursing leadership,10) case reviews	Compliance with insertion practices: 100%. Compliance with dressing maintenance: 80-100% during the intervention period.
McPeake[37] 2012, UK, Central line	Quality improvement: Pros cohort	Jun '08-Dec '10	General ICU (Number of beds NS)	CLABSI rate<br><br>Compliance	ICU nurses	PDSA cycles	AON/ Per month	1) checklist, 2) performance feedback, 3) education, 4) run charts on trolley, 5) Evaluation when compliance with an element was poor 6) checklists for insertion	Jun '08: 67%<br>Dec '10: 100%

bundle									
Mc Namara[38] 2011, USA, Central line bundle	Retrospective study	Oct'08- Apr'10	General ICU (89 beds)	Bundle documentation	Nurses	NS	Lowest level of compliance + item-by-item/ Per month	1) feedback to included units and nursing staff, 2) audit, 3) electronic insertion checklist, 4) time-out procedure	Compliance: Oct '08: 20% - Apr. '10: 80%.
Helmick[39] 2014, USA, Central line bundle	Retrospective review	2009-2011	Multicentre: 5 mixed, 4 medical, 4 surgical and 2 cardiac ICUs.	Mean rates of CRBI	NR	NS	Lowest level of compliance/ Per month	1) Checklists	2009: 95.86% 2010: 98.31% 2011: 97.51% P 0.38
Lawrence[16] 2012, Australia, Ventilator bundle	Pre/post design	Total period: Apr'09-Mar 10. Pre-intervention 6 months, Intervention: 6 months	2 General ICUs, 8 beds	Compliance	Nurses	NS	AON/ Overall: 6 months	1) feedback of compliance rates using a graphical presentation.	Pre intervention (n=164): 65.2% Intervention (n= 151): 67.5% (p-value Chi-square: 0.754)
Morris[40] 2011, UK, Ventilator bundle	Pre/post design	Baseline: Jan '05 - Feb '08. Run in period: Feb '08- Sept '08. Post VAP prevention period: Sept '08 - Aug '09.	Medical & surgical ICU, 18 beds	VAP incidence MRSA rates Duration of MV ICU LOS ICU mortality	Nurses and medical staff	PDSA-cycle	AON/ Overall: 1 year	1) distribution of teaching materials; 2) Education sessions; 3) Feedback on compliance at meetings, by email, with posters; 4) bedside cues; 5) changing the 24h observational chart (checklist); 6) nurse and medical champions.	Post implementation period: 70%
Hawe[41] 2009, UK, Ventilator bundle	Pre/post design	Passive implementation period: Sep '05- feb '07. Active period: 1 Mar '07- 31 Dec '07	Medical & Surgical ICU	Compliance VAP incidence Unit mortality	Medical and nursing staff	NS	AON/ Per month	Passive period: 1) formally adopted as unit policy; 2) laminated copies of the bundle at pt bedside; 3) verbal and written encouragement for its use at ward rounds and other times. Active period: 1) educational intervention: multidisciplinary education meetings; 2) workshops for medical and nursing staff presenting the definition, pathogenesis, epidemiology and risk factors of VAP; 3) Written material for self-study was distributed; 4) assessment of bundle compliance during daily rounds; 5) Feedback of process measurement; 6) feedback of outcomes measurement and organizational change; 7) Barriers affecting delivery of care were identified and iteratively improved.	Passive period: Nov '06: 0% Active period: may '07 48%, Oct '07: 54% (chi square p value: <0.0001)
Bloos[42] 2009, Germany, Ventilator bundle	Pre/post design	Total period: Jun '05-Jun '06. Pre intervention: Jun-Sep '05, Post intervention Mar-Jun'06	General ICU, 50 beds.	Compliance ICU LOS Rates of pneumonia Days on MV ICU survival	ICU nurses and residents	NS	AON/ Overall: 4 months	1) educational meetings: scientific background and techniques of the bundle were taught to all nurses and residents in daily seminars for 2 months; 2) red marks on the wall to indicate a correct semi recumbent position; 3) individually training of nurses or residents if the bundle was not correctly applied (for 2 months); 4) change team (for training staff): consisting of ICU manager, ICU consultant, ICU residents and nurses.	Audit 1: 15%, Audit II: 33,8% (p<0,01)

Al-Tawfiq[43] 2010, Saudi Arabia, Ventilator bundle	Pre/post design	Pre intervention: Jan-Dec '06. Post implementation: Jan '07-Dec '08	Private general hospital, Medical, Surgical, Cardiac ICU 18 beds	Compliance Device utilization ratio VAP rate	Nurses, doctors and respiratory therapists	NS	AON/ Per quarter	1) education of staff; 2) feedback of compliance to team + quarterly feedback of compliance and VAP rates; 3) checklist; 4) audit tool; 5) daily multidisciplinary rounds; 6) team approach to drive and maintain the initiative (including infection control professional, nurse, respiratory therapist, intensivist and chairman of the infection control committee). 7) if non-compliance with an element was detected, the nurse intervened in this process at the time of monitoring process.	First 3 month of implementation period (first quarter 2006): 20%, Last 3 month (fourth quarter 2008) of implementation period 82%.
Jimenez[44] 2009, USA, Ventilator bundle	Pre/post design	Pre intervention 2 weeks. 4 months of non-measurements. Post intervention 2 weeks.	Neuro surgical ICU, 18 beds	Compliance	Nursing staff	NS	AON + Item-by-item/ Per 2 weeks	1) education sessions; 2) weekly feedback on compliance; 3) posters; 4) reminder cards;	Pre education: n= 102, compliance 6 %, Post education, n= 86, compliance 59%
DuBose[45] 2008, USA Ventilator bundle	Pre/post design	Baseline: 1 month. Intervention period 3 month	Single center, trauma ICU	Compliance	ICU staff	NS	Item-by-item measurement/ Per month	1) Quality Round Checklist (QRC) developed by multidisciplinary team; 2) education; 3) multidisciplinary team to enhance implementation; 4) laminated flyers,	Pre-intervention: HOB: 35,2%, PUD prophylaxis 76,2%, DVT prophylaxis (91,2%), Sedation holiday: 78%. Implementation month 3: HOB: 84,5%, PUD prophylaxis 92,3%, DVT prophylaxis 92,8, Sedation holiday: 86%
Berenholtz [46] 2011, USA, Ventilator bundle	Pre/post design	Baseline: 3 months. Post intervention: 0-30 months after implementation	Multi center, 112 mixed, medical, surgical/ trauma, cardiac ICUs	Quarterly VAP rates Compliance	ICU teams	Model for organizational change (i.e. engage, educate, execute, evaluate) Theory of planned behavior	Composite + Item-by-item/ Per quarter	1) local improvement team (ICU director and ICU nurse manager, ICU physician, nurse and senior hospital executive); 2) staff engagement by posting baseline compliance and VAP rates and discussing patients who developed VAP; 3) education (fact sheet, summary guidelines, a slide set en references); 4) daily goal checklist; 5) create protocols and order sets; 6) enlist support from clinicians and family members; 7) feedback: monthly number patients who developed VAP and bundle compliance and quarterly rates of VAP; 8) show reports of other ICUs to compare performance.	Baseline: 1881/5789 (32%) Intervention period: 48% Post intervention quarterly: 0-3 months: 50,%, 28-30 month: 84%
Lim[47] 2013, Taiwan, Ventilator Bundle	Pre/post design	Jan'06-Mar'13	Tertiary referral medical center. Surgical ICU 63 beds	ICU utilization, ventilator utilization, VAP incidence, compliance	Nurses, doctors, respiratory therapists	NS	AON/ Overall: 1 year	1) education; 2) posters; 3) checklists; 4) standardizing medical interventions and equipment.	Post intervention (after re-education): doctors: 99%, nurses: 89.3%, respiratory therapists: 84%.
Mukhtar[48] 2014, Egypt,	Pre/post design	Mar'11-Jun'12	University hospital. Surgical ICU:	- VAP rates caused by MRSA - compliance	Nurses	NS	AON/ Overall: 1 year	1) education; 2) rewards to motivate nurses for perfect fulfilment of the VCB.	Compliance ranged from 60%-70%

Ventilator bundle			8 beds						
Eom[49] 2014, South Korea Ventilator bundle	Quasi-exp: Pre/post design	Pre-intervention: Jul'11-Feb'11, Post intervention: Mar '11- Jun '11	Multicentre, 6 University hospitals. 196 ICU beds	VAP rates	23 doctors, 318 nurses	NS	AON + Item-by-item/ Overall: 4 months	1) Education on VAP and VAP bundle; 2) checklists; 3) regular feedback on compliance; 4) nurses intervened at the time of monitoring if non-compliance with a bundle element was detected.	Overall compliance (without CASS): Pre intervention: 41.1%, post intervention: 71.8%.
Hamishehkar [50] 2014, Iran, Ventilator bundle	Observational study Pre-post design	Pre-education phase: 45 days, education phase, post education phase: 1 month after the education phase ended.	Multicentre, 4 university affiliated hospitals. 3 surgical, 2 general, 2 pulmonary, 1 neurosurgical, 1 neuro ICU	Compliance	143 nurses: 127 (89%) females; age (mean±sd): 33±5; workexp: (mean±sd): 10±4yrs	NS	AON, Mean compliance/ Time period not clear	1) education, educational pamphlets containing results of VAP bundle compliance in each ICU and VAP bundle control guidelines and delivered them to every nurse whose practice was evaluated.	Pre-education (N=294): 36.5%, post education (N=258) 41.2%.P > 0.05
Malouf Todaro[51] 2013, USA, Ventilator bundle	Quality improvement: pre/post design	Pre intervention 2 months. Post intervention 6 months	Medical and surgical ICU, 24 beds.	Compliance	Nurses	NS	AON/ Overall: 6 months	1) education; 2) use of electronic checklists and developed in collaboration with focus groups of ICU nurses and physicians.	Pre intervention: 5/137 (3,7%) Post intervention: 464/504 (92.1%)
Rello[52] 2013, Spain, Ventilator bundle	Prospective cohort	Baseline: 3 months. Intervention period: 16 months	Multicenter, 5 general ICUs	Compliance VAP rates ICU length of stay Duration of MV	ICU teams	NS	Lowest level of compliance/ Overall: 6 months	1) feedback on VAP rates and compliance in the form of posters and feedback to improvement team; 2) local ICU improvement teams (incl medical leader and nurse manager); 3) posters; 4) cards and brochures to educate staff; 5) education; 6) checklist	Compliance rate complete bundle: 20%
Berenholtz [53] 2004, USA, Ventilator bundle	Prospective cohort	4 Mar '02-29 Apr '02 (8 weeks)	Tertiary hospital. Surgical ICU, 20 beds (14 in use).	Percentage of ventilator days per week Compliance	Nursing staff	NS	Composite / Per week	1) survey to identify barriers of compliance, 2) education to provide a summary of the evidence; 3) checklist to be completed daily during daily rounds; 4) billboard within ICU to highlights the project and post the performance; 5) interdisciplinary team to lead Quality Improvement effort; 6) discuss performance during daily rounds; 7) adding topics to the agenda at monthly performance improvement meetings	Before start intervention: 30%, after intervention: 96% (p<0.001)
DePalo[54] 2010, USA Ventilator bundle	Prospective cohort	Data reported from 1 Jan '06 - 30 Jun '08. Baseline period: 1 Jan '06- Mar '06.	Multi center, 11 hospitals, 23 ICUs, 263 beds	VAP rates	Not reported	Patient safety program	AON/ Per quarter	1) patient safety based program: educate staff on science of safety, learning from defects and implementing work tools and empowered to stop procedures. 2) Education, 3) feedback of infection rates 4) survey culture: culture assessment tool used to survey the culture of each ICU, administered at the start of the project and annually thereafter. 5) engagement, 6) empowering nurses to stop physicians who do not follow the checklist 7) implementation teams, 8) checklists	Quarter 1 '06: 60%, Quarter 2 '08: 78% (p<0.0001)

Al-Thaqafy[55] 2014, Saudi Arabia, Ventilator bundle	Prospective cohort	Jun'10-Dec'13	Tertiary hospital, medical, surgical and trauma ICU, 21 beds.	Compliance<br>VAP rates<br>Association between compliance and ventilator utilization	Nurses and physicians. Nurse to patient ratio is 1:1	NS	AON/<br>Per quarter	1) Periodic educational and training sessions; 2) daily multidisciplinary rounds; 3) no blame policy	Compliance Q2 2010: 86% to 99% in Q4 2013
Hatler[56], 2006, USA, Ventilator bundle	Quality improveme nt: prosp cohort	15 months	Medical ICU, 8 beds	Compliance<br>VAP rates	Nursing staff	Theory of planned behaviou r	AON/<br>Per month	1) education; 2) one-page document detailing new strategies to address prevention of VAP; 3) regular feedback infection rates and rates of adoption; 4) multidisciplinary project team to monitor progress and to make needed changes during implementation; 5) daily rounds; 6) daily goals were posted on white boards in patient rooms; 7) charts of expected activities were posted in each patient room; 8) reinforcement by giving movie tickets to nurses; 9) when sign. change was accomplished, staff members, managers and administrators celebrated with prizes; 10) newsletters	Begin: 73%, end of the intervention period: 98.6% ($p<0.001$)
Esmail[57] 2008, Canada, Ventilator bundle	Quality improveme nt: pros cohort	Start quality improvement project in 2002-jun'07	ICUs, 38 beds	Compliance<br>VAP incidence	Critical care team	PDSA-cycle	AON/<br>Per month	1) educational sessions; 2) feedback of performance and VAP rates; 3) creation of a multidisciplinary team to work on VAP prevention; 4) family participation: checking bundle compliance;<br>5) checklists; 6) signs for HOB; 7) HOB alarm on all new beds; 8) newsletters; 9) review a VAP case at meetings;10) posters; 11) audits	Modified bundle: Sept '04: 45% Jun '07: 100%.
Young-quist[10] 2007, USA, Ventilator bundle	Quality improveme nt: prosp cohort	Jan '03-May '04	2 hospitals. Medical, surgical and cardiovascula r surgery ICU, 40 beds	Compliance<br>VAP rates	ICU staff	NS	AON/<br>Per month	1) fact sheets; 2) posters with VAP rates; 3) written communication to remind and motivate ICU staff; 4) education of nurses in July '03; 5) HOB added to preprint ventilator orders: Aug'03; 6) reminder signs for HOB signs at bedside: Sept '03;<br>7) daily multidisciplinary rounds with emphasis on bundle: Jun '04; 8) monthly feedback of compliance audits	Mercy hospital: May '03: 0%, Jun '04: 100%.<br>Unity hospital: May '03: 0%, Jun '04: 89%.<br>Mean compliance: 94.5%
Bonello[35] 2008, USA Ventilator bundle	Quality improveme nt: pros cohort	Jan '05-Jun '06	Multicenter, 8 hospitals, 12 ICUs, 95 beds	Compliance<br>VAP rates	ICU care providers	PDSA-cycle	AON/<br>Per quarter	1) educational symposia; 2) interdisciplinary team rounds; 3) checklist at patient bedside; 4) multidisciplinary implementation teams	VAP bundle: First 3 months: 50%, final 3 months: 82%
Miller[58] 2010, USA, Ventilator bundle	Quality improveme nt: prosp cohort	1 Jan '06-30 Apr '08.<br>VAP bundle implementation began in 2007	University hospital, Trauma ICU, 14 beds	Infections rates	Nursing staff	NS	AON/<br>Per month	1) feedback compliance and VAP rates; 2) online checklist for compliance; 3) real time dashboard view of compliance. For each element of the bundle an indicator is displayed showing status with regard to the desired intervention; 4) education; 5) auditors to monitor and ensure compliance; 6) modifications to clinical processes; 7) nursing leadership periodically audited compliance	Compliance with VAP bundle: Aug '07: 12%, Apr '08: 70%
Bukhari[59] 2012, Saudi Arabia, Ventilator bundle	Prospective longitudinal	Jan'10-Dec'10	Medical and surgical ICU, 18 beds	Compliance<br>Rate of pneumonia<br>Days on MV<br>Lengths of ICU stay	Nurses and residents	NS	AON/<br>Per month	1) weekly seminars to staff; 2) checklists; 3) visitors and family education was given and if the bed is not in required position they should inform the treating nurse; 4) daily multi-disciplinary rounds to assess compliance and discuss bundle elements; 5) two surveys entitled:	Whole period: 78,9%<br>Jan '10: 30%, Dec'10: 100%.

								knowledge, attitude and practice of ICU staff on VAP bundles were conducted; 6) respiratory therapists worked collaboratively with nurses; compliance monitoring 8) auditing VAP and compliance; 9) VAP rates presented	
Marra[60] 2009 and Caserta[61] 2012, Brazil Ventilator bundle	ITS	Phase 1: Mar'01-Dec'02 (implementation HOB + vent circuits) Phase 2: Jan'03-Dec'06 (implementation HOB, vent circuits, heat and moisture exchange). Phase 3 implementation ventilator bundle: Jan'07-Sept '08 Oct '08-Dec '10	Medical and surgical ICU, 38 beds	Compliance In-hospital mortality VAP incidence	Nurses, doctors and respiratory therapists	NS	AON/ Per month	1) feedback on compliance; 2) posters on compliance rates and VAP rates; 3) performance monitoring each weekday and 4) intervene in this process at the same time that performance monitoring was occurring at the bedside if non-compliance was detected, 5) brief presentation of VAP to the staff, 6) encourage participation in the "ventilator bundle- getting to zero" program.	Phase 3: Apr '07: 57% - Sept 100% Oct '08: 95%, End: Dec '10: 99%.
Zaydfudim [62] 2009, USA, Ventilator bundle	ITS	Jan '05-Jul '08	Tertiary University hospital. Surgical ICU, 21 beds	VAP rates Compliance	ICU team	NS	AON/ Per month	1) electronic dashboard: displays compliance with the ventilator bundle parameters for each ventilated patient at timed intervals for each measure; 2) compliance with patient-level dashboard parameters were reviewed twice daily; 3) physician and nursing leadership received daily compliance reports.	Aug '07: 39% - Jul '08: 89% (p<0.001)
Crunden[63] 2005, UK, Ventilator bundle	Retrospective study	Group A: Jan '02 to14 Dec ''02. Group B: 15 Dec'02 to 31 Dec '03	Single center, 6 ICU beds	Compliance Length of MV ICU LOS	ICU teams	NS	Composite/ Per month	1) checklists; 2) daily rounds: every day on the round the staff assess the appropriateness of implementing each element of the bundle per patient; 3) education	Audit 1 month. Baseline Oct '02: n=21 bundle compliance: 21%, July '03: n=24 bundle compliance 79,1%
Helmick[39] 2014, USA, Ventilator bundle	Retrospective review	2009-2011	Multicentre: 5 mixed, 4 medical, 4 surgical and 2 cardiac ICUs.	Mean rates of VAP	Not reported	NS	Lowest level of compliance/ Per month	1) Checklists	2009: 86.2% 2010: 81.1% 2011: 89.8% P 0.76
Guiliano[64] 2011, USA Sepsis resuscitation/ management bundle	Pre/post design, pilot	Mar 1, '06 – Aug 5, '08. The 2 phases of the study were completed between this time periods. Phase 1= before intervention, phase 2 after	2 hospitals	-compliance -time to completion -time to administration	ICU nurses	NS	Mean percentage of the completion of the bundle/ Time period not clear.	1) education; 2) protocol watch: this is a proprietary CDSS application for bedside monitoring of patients that was designed by Philips Healthcare to assist clinicians in implementing the SSC guidelines.	Resuscitation bundle (%, mean (SD)): Phase 1: 57.6 (19.8), phase 2: 68.2 (20), p0.003 Management bundle: (%, mean (SD)): Phase 1: 84.5 (19) Phase 2: 86.8 (17), p 0.48

		intervention							
Ferrer[65] 2008,<br>Spain,<br>Sepsis resuscitation/ management bundle	Pre/post design	Pre intervention: 2 months: Nov-Dec'05<br>Post intervention: 4 month: March-Jun'06<br>Long term follow up 1yr later on a subset of 23 ICUs: Nov-Dec'06	Multicenter, Medical and surgical ICUs, 59	-hospital mortality<br>-adherence<br>-ICU mortality<br>-28-day mortality<br>-HLOS<br>-ICU LOS	Physicians and nurses	NS	AON + Item-by-item/<br>Overall: 4 months	1) educational program: training physicians and nursing staff in definitions of severe sepsis and septic shock, their early recognition and the treatment included in the guidelines; 2) posters; 3) pocket cards; 4) audit and feedback 5) the principal investigator acted as local champion; 7) creation of local multidisciplinary teams: they reviewed the pre-intervention performance data and shared ideas about the process improvement goals and strategies.	Resuscitation bundle:<br>Pre intervention: n=854: n, (%) [CI 95%]: 45 (5.3%), [BI 4-7] p<0.01, Post intervention: 147 (10), [BI 8-12].<br>Management bundle: pre intervention: n=1465: 93 (10.9), [9-13] Post intervention: 230 (15.7), [14-18]. P <0,001.
Castellanos-Ortega[66] 2010,<br>Spain,<br>Sepsis management bundle	Quasi exp: pre/post design	Pre intervention baseline: 12 months: Jan '04-May '05.<br>Implementation period: 3 months: June - Aug '05.<br>Post intervention: 3 yrs: Sept '05-Aug '08.	Single center, Medical and surgical ICU, 30 beds	-in-hospital mortality<br>-LOHS<br>-ICU mortality<br>-compliance	Physicians and nurses	NS	AON + Item-by-item/<br>Overall: 3 years	A hospital wide implementation program.<br>1) educate staff in recognizing severe sepsis and septic shock + intervention included in guidelines; 2) audit; 3) feedback; 4) posters; 5) pocket cards; 6) lectures; 7) sepsis profile: optional tool to facilitate an early diagnosis of sepsis and severe sepsis.	The management bundle in only implemented within the ICU.<br>24h-management bundle: Historical group (baseline): n=96: 0 (0), Intervention group: n=384: 5 (1.3%).
Lefrant[67] 2010,<br>France<br>Sepsis bundle	Quality improvement: pre/post design	1 Jan - 30 jun'06 (observational period), 1 Jul-31 dec'06	Multicenter, 15 ICUs	-28-day mortality<br>-compliance	Nurses, physicians and residents	NS	AON + Item-by-item/<br>Overall: 6 months	1) educational meetings; 2) distribution of educational materials; 3) posters and pocket cards with bundle; 4) video transmission meeting	Baseline (observational period): 1/230 (0.5%), Interventional period: 9/215 (4%).
Silverman [68] 2011,<br>USA,<br>Sepsis bundle	NS<br>Pre/post design	Phase 1: historic cohort<br>Phase 2: 2006: bundle implementation<br>Phase 3: from Sept'08 bundle plus implementation	Single center, Surgical ICU	-mortality rate<br>-costs	NR	NS	AON/<br>Overall: 2 years	Phase 2: 1) education of staff; 2) multidisciplinary sepsis team created: one member rounded 2-4 times per week to ensure all appropriate patients were on the bundle. Team developed tools and order sets to facilitate the use of the bundle.<br>Phase 3: addition of an intensivist in 2008: Surgical ICU care team created	Phase 2: Bundle group: 19/186 (10%), Phase 3: Bundle plus group: 47.6/68 (70%)
Memon[69] 2012,<br>Saudi Arabia,<br>Sepsis resuscitation bundle	Quasi experimental prospective study	Pre intervention: 15 **months**: Jan '08-March 09.<br>Intervention period: 3 month:Apr-Jun'09<br>Post intervention: 24 month: Jul '09-Jun '11	Medical and surgical ICU, 10 beds	-overall compliance<br>-30-day mortality<br>-ICU mortality<br>-ICU LOS<br>-LOHS	Physicians, nurses and respiratory therapists.	NS	AON/<br>Overall: 2 years	1) educational lectures; 2) special Task Force Teams; 3) they developed a written protocol to prepare the sepsis bundle for the management of patients with severe sepsis or septic shock	Pre intervention (historic group): 5/99 (5.1%)<br>Post intervention: 47/199 (23.6%)
Schramm[70]	Prospective	Overall: jan'07-	Single center,	-compliance	ICU	NS	AON + Item-by-	1) educational meetings/teaching session; 2) distribution	Baseline: 34/268

2011, USA, Sepsis resuscitation bundle	intervention al cohort study	sept'09. Baseline: jan'07-28dec'07 Weekly feedback: 29dec'08-26sept'08 Inzet SRT: 27sept'08-30sept'09	Medical ICU, 24 beds	-mortality	healthcare providers		item/ Overall: 1 year	of educational materials; 3) daily auditing and weekly feedback; 4) multidisciplinary bedside response teams (SRT)	(12.7%), Period of weekly feedback: 107/284 (37.7%), Sepsis Response Team period: 232/432 (53.7%). P<0.001
Kim[71] 2012, South Korea, Sepsis resuscitation/ management bundle	Prospective observation al study.	July '09	Multicenter, Medical and surgical ICU, 28 ICUs	-overall hospital mortality -28-day mortality -ICU mortality -ICU LOS -duration of MV	Critical care personnel	NS	AON/ Per month	1) full time intensivists available by turns of duty for 24h and nurse to patient ratio of 1:2	n=251. Complete Resuscitation bundle: 31/251 (12.35%), Management bundle: 39/251 (15.54%)
Laguna Perez [72] 2012, Spain, Sepsis resuscitation/ management bundle	Quasi experiment al prospective study	Control group: Jun'08-Jul'09 Intervention group: Oct'09-March'10	Single center	-compliance -survival rates -LOHS	NR	NS	Item-by-item/ Overall: 6 months	1) education and training programs; 2) posters with protocol algorithms and diagrams were made; 3) developing reference guides and made available at nursing controls and on intranet	6- hour bundle: control group: element 1. 55/84 (64,7%), 2. 44/84 (52.4), 3. 33/84 (40.7%), 4, 35/84 (41.7%) 16/84 (19%), 11/84 (13.1%), 60/84 (71.4%) Intervention group: 1, 30/41 (60%), 2, 29/41 (70.7%), 3, 17/41 (41.5%), 4, 32/41 (78%), 5, 22/41 (53.6%), 6, 18/41 (43.9%), 7, 32/41 (78%). 24-hour bundle: Control group: 1. 26/85 (30.5%). 2, 39/76 (51.3%), 3, 16/84 (19%), 4, 6/58 (10.3%) Intervention group: 1. 40 (40), 2. 22/36 (61.1%), 3, 32/40 (80%), 19/23 (82.6%).

CL: Central Line, CLABSI: Central Line Associated Blood Stream Infection, CRBSI: Catheter Related Bloodstream Infections, PDSA-cycle: Plan Do Study Act-cycle, NS: Not Stated, MV: Mechanical ventilation, LOS: Length of Stay, ICU: Intensive Care Unit, SSC: Surviving Sepsis Campaign, MRSA: Methicillin Resistant *Staphylococcus Aureus.*
