# Supplementary material for: What are effective strategies for the implementation of care bundles on ICUs: a systematic review
Source: Implement Sci. 2015 Aug 15;10:119. doi: 10.1186/s13012-015-0306-1 (PMC4536788; doi:10.1186/s13012-015-0306-1)
Supplement: Additional file 4: — Bundle elements. (PDF 150 KB) [file 13012_2015_306_MOESM4_ESM.pdf]

Additional file 4 Bundle elements

Central line bundle	Exline et al. Insertion bundle	Longmate et al, Insertion bundle	McPeake et al. Insertion bundle	Hocking et al, Insertion bundle	Khalid et al., insertion bundle	Khalid et al., maintenance bundle	Hocking et al. Maintainance	Exline et al. Maintainance	Marra et al, Central line bundle	Bonello et al, Central line bundle	Jeong et al, Central line bundle	Richardson et al, Central line bundle	Helmick et al. Central line bundle	Sacks et al. Central line bundle	Render et al, Central line bundle	McNamara et al. Central line bundle
Hand hygiene			x	x	x				x	x	x	x	x	x	x	x
Maximal barrier precaution	x	x	x	x	x				x	x	x	x	x	x	x	x
Full body drape				x												x
Chlorhexidine skin antisepsis	x	x	x	x		x			x	x	x	x	x	x	x	x
Optimal catheter site selection, with avoidance of femoral vein	x	x	x	x	x				x	x	x	x	x		x	
Daily review of line necessity with prompt removal of unnecessary lines						x	x	x	x	x		x	x	x	x	
PIC placement with ultrasound guidance to avoid CVC placement and to facilitate removal.	x															
Removal within 24h of all CVC's placed emergently								x								
Use of a checklist for insertion		x	x						x							
Time out note	x															x
Infusion IV nutrition via a dedicated lumen																
Daily checking of central line site for inflammation							x									
Cleaning of all ports with 2% chlorhexidine and 70% alcohol prior to assessing CL							x									
Chlorhexidine impregnated dressings and/or AB impregnated CLs for high risk pt.							x									
Prevention measure in use e.g. biopatch disc or chlorhexidine gluconate (CGH) dressing																
Intervene at the same time when non-compliance was detected (=time out procedure?)									x							
Sterile techniques while inserting the CL and applying the dressing				x												
Dressing with Chlorhexidine gluconate material								x								
Preferential use of the subclavian vein														x		
Patient hygiene					x											
Catheters coated internally with silver sulfadiazine and chlorhexidine acetate					x											
Chlorhexidine mouthwash for oral care						x										

Ventilator bundle	Hatler et al, 2006	Malouf Todaro et al, 2013	Lawrence et al, 2012	Morris et al, 2011	Hawe et al, 2009	Bloos et al, 2009	Rello et al, 2013	Bukhari et al, 2012	Marra, 2009/Caserta, 2012	Esmail et al, 2008	Youngquist et al, 2007	Zaydfudim et al, 2009	Al-Tawfiq et al, 2010	Bonello et al, 2008	Miller et al, 2010	DePalo et al, 2010	Jimenez et al, 2009	Dubose et al, 2008	Berenholtz et al, 2011	Crunden et al, 2005	Berenholtz et al, 2004	Helmick et al., 2014	Eom et al., 2014	Hamishehkar et al. 2014	Lim et al., 2013	Mukhtar et al. 2014	Al-Thaqafy et al., 2014
Elevation of the head of the bed (HOB)	x	x	x	x	x	x		x	x	x	x	x	x	x	x	x	x	x	x	x	x	x	x	x	x	x	x
Daily sedation vacation		x	x	x	x		x					x	x	x	x	x		x	x		x	x				x	
Assessment of readiness to extubate				x	x								x	x								x					
Peptic ulcer disease prophylaxis	x	x	x			x		x	x		x		x	x	x	x	x	x	x	x	x	x	x		x	x	x
Deep Venous Thrombosis prophylaxis	x	x	x			x		x	x		x		x	x	x	x	x	x	x	x	x	x	x		x	x	x
Daily oral care		x		x	x		x					x			x							x	x	x	x	x	x
Hand hygiene		x					x																	x			
Intra cuff pressure							x																				
Tubing management					x		x																				
Subglottic suctioning					x					x		x			x									x			
Preferential use of oral vs nasal tubes for access to the trachea or stomach										x																	
Spontaneous breathing trials												x															
Lung protective ventilation in patients with ALI						x																					
Daily sedation vacation and assessment of readiness to extubate	x							x	x	x	x				x	x	x		x	x					x		x
Dental care												x															
Continuous aspiration of subglottic secretions (CASS)												x											x				
Maintain endotracheal cuff pressure (ETCP)																								x			
Use of a closed suction system																								x			
Use of a endotracheal tube with a separate dorsal lumen																								x			
Spontaneous breathing trial																										x	

Sepsis bundle	Memon et al, 2012	Laguna Perez et al, 2012	Kim et al, 2012	Guiliano et al, 2011	Schramm et al, 2011	Ferrer et al. 2008	Castellanos-Ortega et al, 2010	Silverman et al, 2011, Sepsis bundle	Lefrant et al, 2010, Sepsis bundle	
**Sepsis management bundle**										
Appropriate use / no use steroids		x		x		x	x	x		
Appropriate use / no use Xigris		x		x		x	x	x	x	
Maintain adequate glycemic control		x	x	x		x	x	x	x	
Appropriate management of inspiratory plateau pressures (IPP)		x	x	x		x	x	x		
Low tidal volume									x	
**Sepsis resuscitation bundle**										
Serum lactate measured	x	x	x	x	x	x		x		
Blood cultures obtained prior to antibiotic administration	x	x	x	x	x	x		x	x	
Improve time to broad spectrum antibiotics	x	x	x	x	x	x		x	x	
Apply vasopressors for ongoing hypotension			x		x					
Maintain adequate central venous pressure	x	x	x	x		x		x		
Intravenous fluids delivered	x	x	x		x			x		
Maintain adequate central venous oxygen saturation	x	x	x	x		x		x		
Achieve and maintain mean arterial pressure ≥65 mmHg	x								x	
Appropriate red cell blood transfusion										
Low doses of corticosteroids when norepinephrine requirements > 6h										
Fluids resuscitation / vasopressors (if appropriate)				x		x		x		
Assessment of CVP and ScvO2 within first 6h										
Blood pressure ≥90 mmHg in case of hypotension		x								
