# Supplementary material for: What are effective strategies for the implementation of care bundles on ICUs: a systematic review
Source: Implement Sci. 2015 Aug 15;10:119. doi: 10.1186/s13012-015-0306-1 (PMC4536788; doi:10.1186/s13012-015-0306-1)
Supplement: Additional file 5: — Effects on compliance. (PDF 115 KB) [file 13012_2015_306_MOESM5_ESM.pdf]

Additional file 5 Effects on compliance

Subset 1

In the first subset, studies are included when pre/post designs were used, methodological quality scored ”fair” or “good”, and when compliance was calculated by using the all-or-none approach. The blue bar within the figures represents the 95% compliance level, thus the level of compliance which should have been achieved.

Figures 1 to 3:

- Overall, 19 studies were included in this first subset, describing the compliance of 21 care bundles.
- Central line bundle: 5 studies included, describing 6 care bundles
- Ventilator bundle: 10 studies included, describing 10 care bundles
- Sepsis bundle: 4 studies included, describing 5 care bundles.

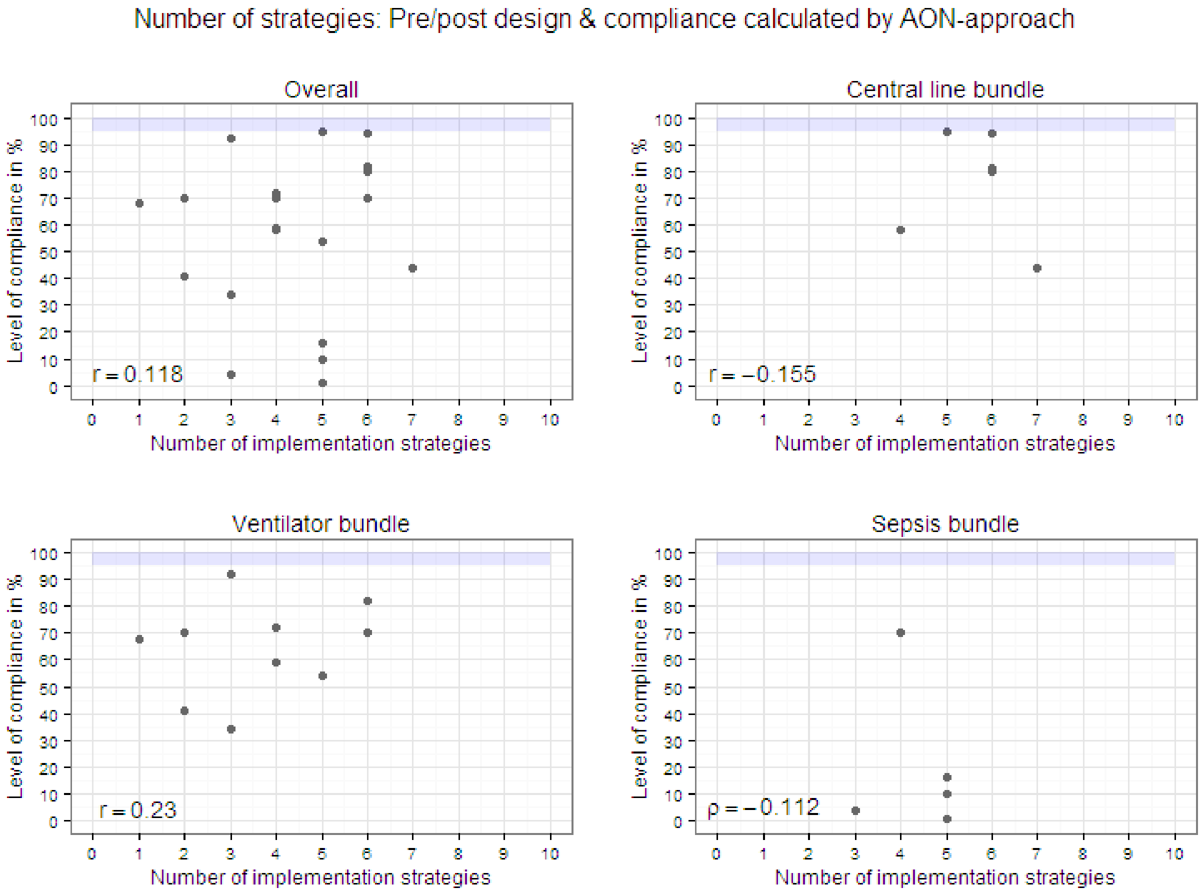

Fig. 1. Number of implementation strategies and compliance levels

Figure 1 shows that overall, there is no relation between the compliance level and the number of implementation strategies used (r = 0.118, 95% CI. -0.331 to 0.523, p = 0.612), neither for each bundle separately: central line bundle: r = -0.155, 95% CI. -0.859 to 0.751, p = 0.769; ventilator bundle: r = 0.230, 95% CI.-0.467 to 0.751, p = 0.522; sepsis bundle: ρ = -0.112, p = 0.858.

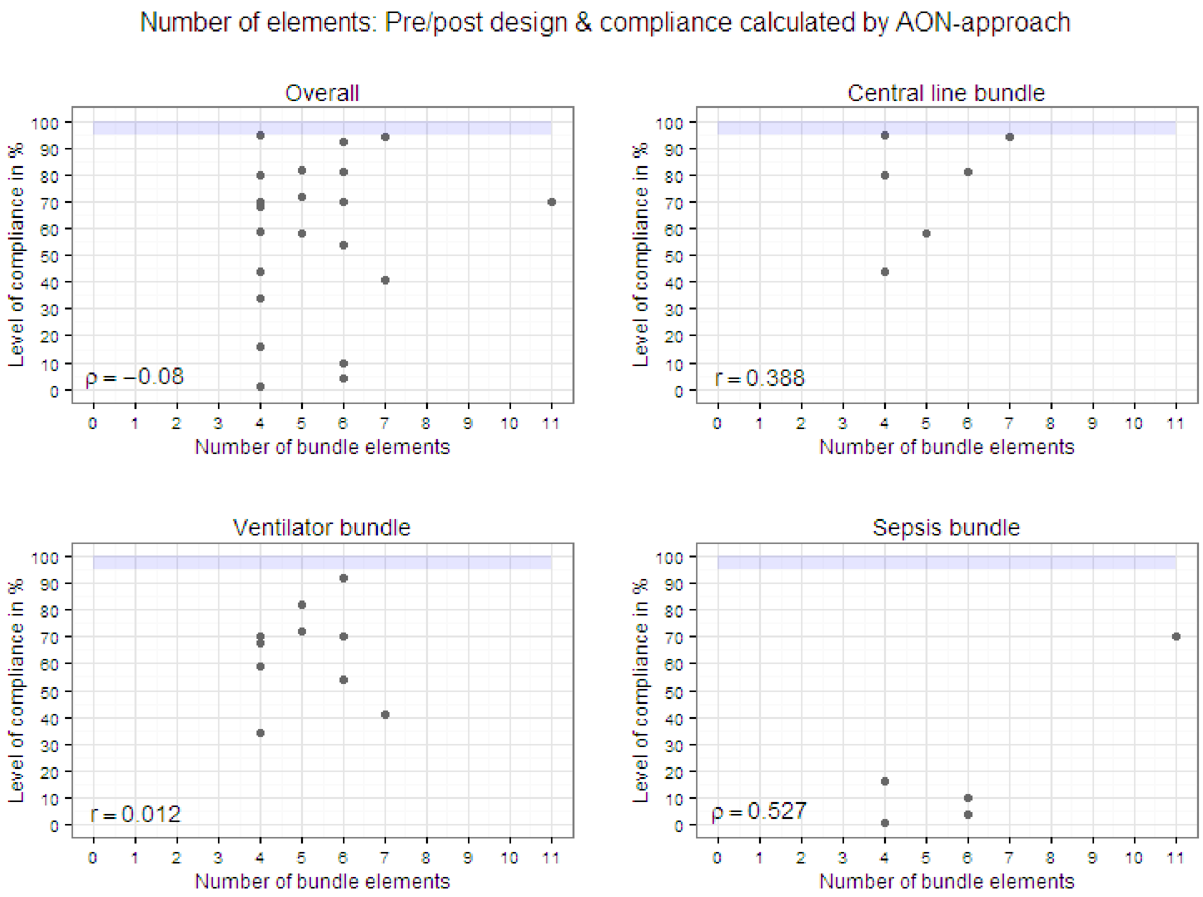

Fig. 2. Number of bundle elements and compliance levels

Overall, there is no relation between the number of bundle elements and the level of compliance (ρ = 0.140, *p* = 0.545), neither for each bundle separately: central line bundle: *r* = 0.388, 95% CI. -0.618 to 0.912, *p* = 0.447; ventilator bundle: *r* = 0.016, 95% CI.-0.620 to 0.639, *p* = 0.965; sepsis bundle: ρ = 0.527, *p* = 0.362.

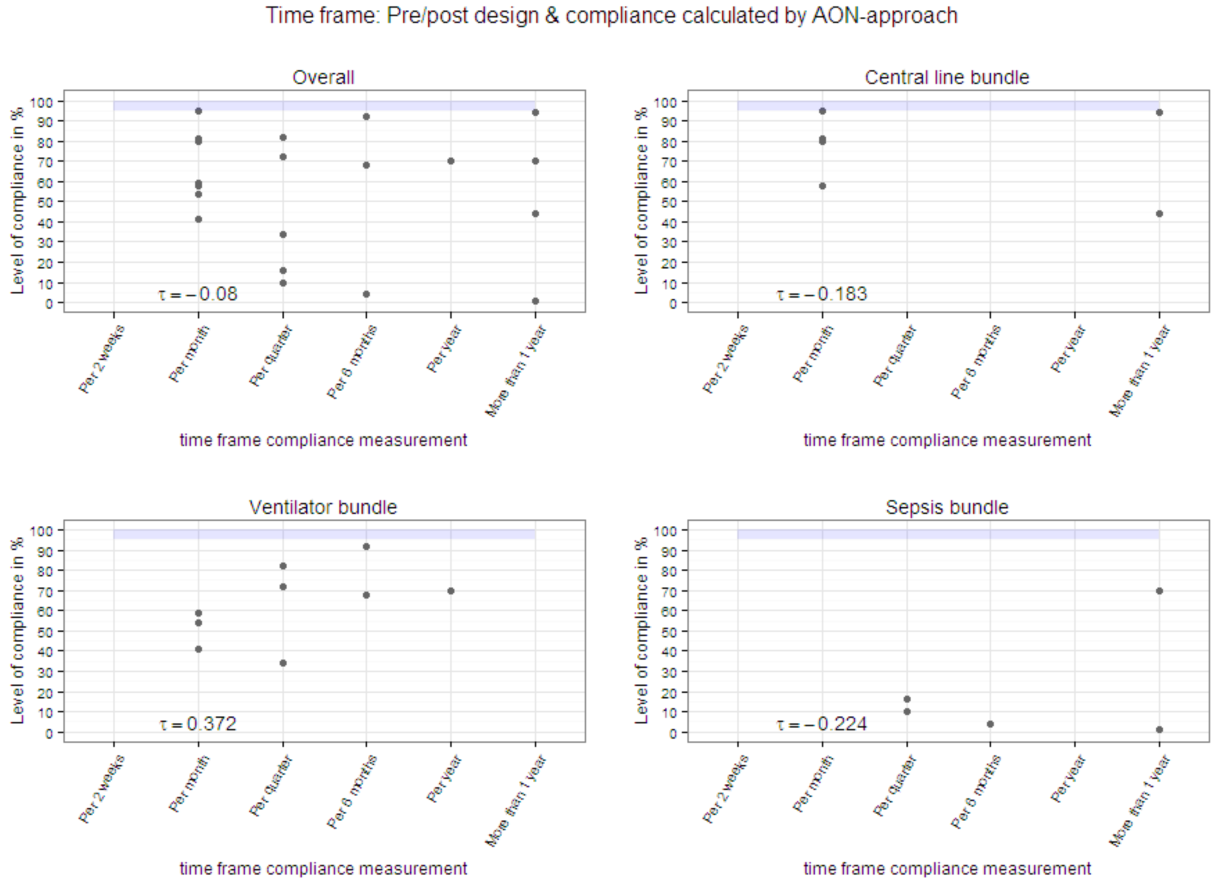

Fig. 3. Relation between compliance and time frame in which compliance is measured

Overall, there is no relation between the time frame and compliance (τ = -0.080, p = 0.639), neither for each bundle separately: central line bundle: τ = -0.183, p = 0.643; ventilator bundle: τ = 0.372, p = 0.162; sepsis bundle: τ = -0.224, p = 0.602).

Subset 2:

In the second subset, studies are included when prospective cohort designs were used, methodological quality scored ”fair” or “good”, and when compliance was calculated by using the all-or-none approach. The blue bar within the figures represents the 95% compliance level, thus the level of compliance which should have been achieved

Figures 4 to 6:

- Overall, 10 studies are included in this subset, describing the compliance of 12 care bundles.
- Central line bundle: 1 study included, describing the compliance of 2 care bundles
- Ventilator bundle: 5 studies included, describing 5 care bundles
- Sepsis bundle: 4 studies included, describing 6 care bundles.

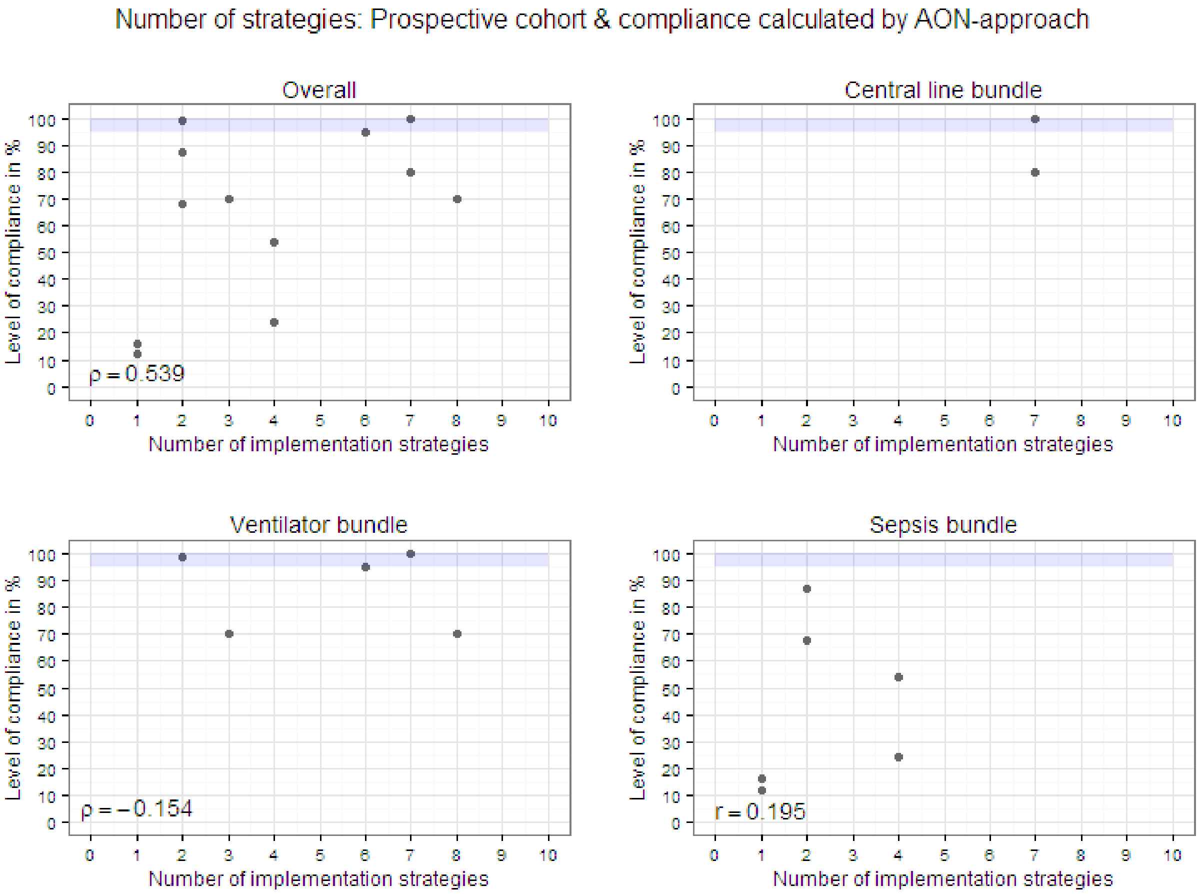

Fig. 4. Number of implementation strategies and compliance levels

Figure 4 shows that there is no relationship between the number of implementation strategies and the level of compliance ($\rho = 0.539$, $p = 0.057$). Correlation coefficients could not be determined for the central line bundle, due to the small number of observations. Ventilator bundle: $\rho = -0.154$, $p = 0.805$; Sepsis bundle: $r = 0.195$, 95% CI. -0.732 to 0.870, $p = 0.711$.

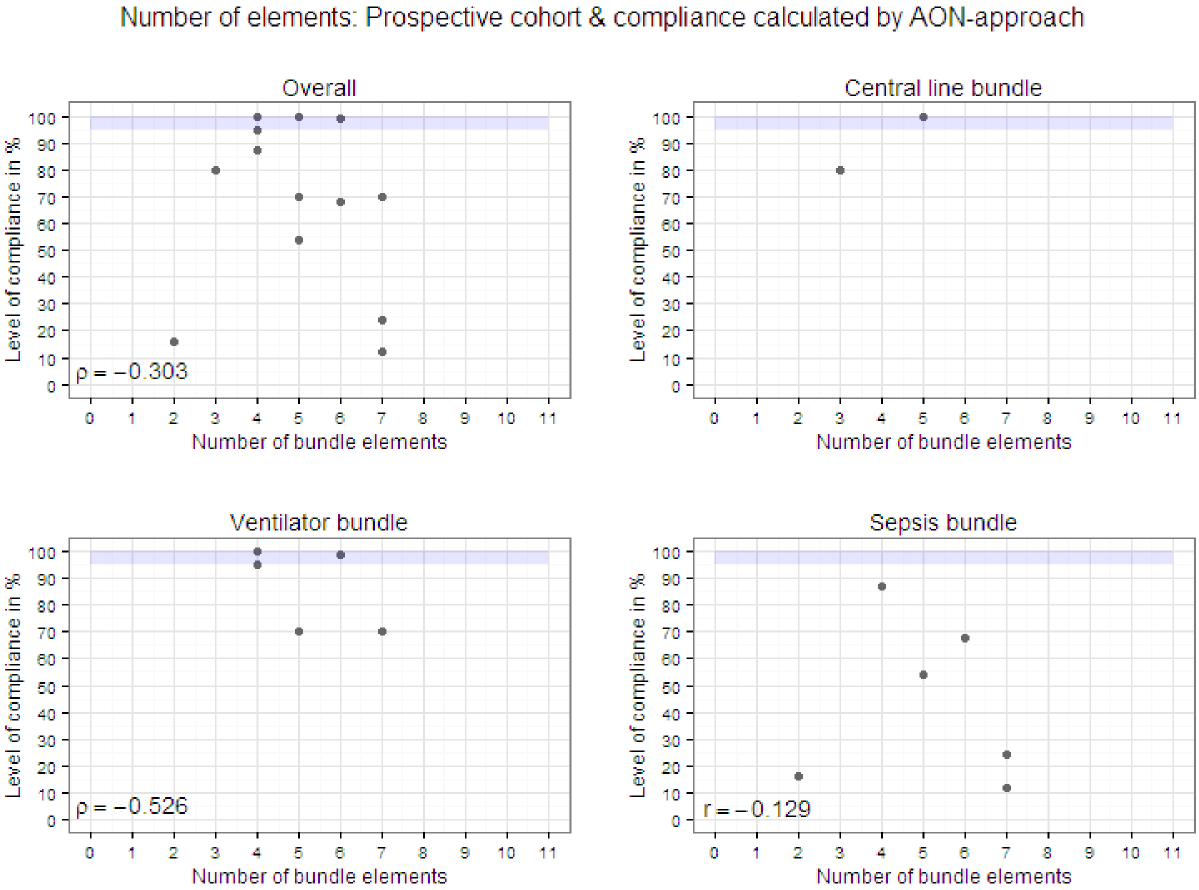

Fig. 5. Number of bundle elements and compliance levels

There is no relationship between the number of bundle elements and the level of compliance (ρ = -0.303, p = 0.314). Correlation coefficients could not be determined for the central line bundle, due to the small number of observations. Ventilator bundle: ρ = -0.526, p = 0.362; Sepsis bundle: r = -0.129, 95% CI. -0.851 to 0.762, p = 0.808.

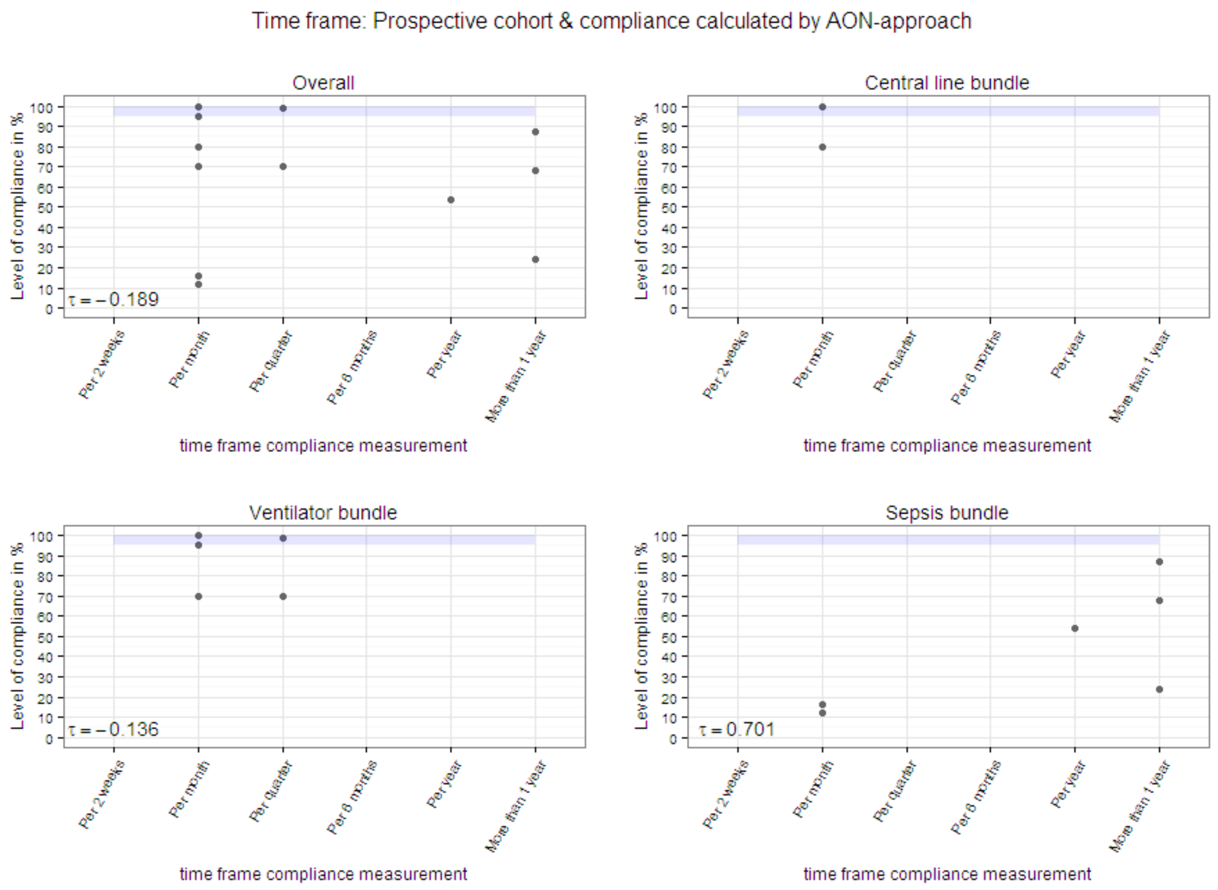

Fig. 6. Relation between compliance and time frame in which compliance is measured.

For the ventilator bundle as well as the central line bundle, compliance is mostly calculated per months and in some studies quarterly. For these studies relatively high compliance levels are measured. Only for the sepsis bundle long time frames are used in which compliance is calculated. However, overall, there is no relation between the time frame and compliance (τ = -189, p = 0.417), neither for each bundle separately: ventilator bundle: τ = -0.136, p = 0.767; sepsis bundle: τ = 0.701, p = 0.064). Correlation coefficients could not be determined for the central line bundle, due to the small number of observations.
